# A web modular AI interface for brain tumor MRI classification using deep learning models

**DOI:** 10.1186/s42490-026-00123-w

**Published:** 2026-10-23

**Authors:** Jennifer Hamalaya, Hadi Fayad, Tarraf Torfeh, Sandy Rihana

**Affiliations:** 1 Department of Engineering (Biomedical Engineering), Holy Spirit University of Kaslik, Beirut, Lebanon; 2 Hamad Medical Corporation, Doha, Qatar

**Keywords:** Brain tumor classification, Magnetic resonance imaging (MRI), Deep learning, Convolutional neural networks (CNN), Glioma, Meningioma, Pituitary tumor, Vgg, Xception

## Abstract

In order to improve patient outcomes and treatment processes, early and precise diagnosis of brain tumors is crucial. Despite the strong performance shown by deep learning models in classifying brain tumors from MRI scans, integrating these models into user-friendly clinical workflows remains challenging. This study presents a web-based AI platform designed for brain tumor classification using MRI images. The platform enables users to upload MRI scans, apply standardized preprocessing, select between different deep learning models, and receive real-time predictions. A custom convolutional neural network (CNN), pretrained VGG19, and pretrained Xception were evaluated using a publicly available T1-weighted contrast-enhanced brain MRI dataset of approximately 7,000 images across four classes: glioma, meningioma, pituitary tumor, and no tumor. The dataset lacks patient demographic and clinical metadata, limiting assessment of clinical generalizability. The models achieved test accuracies of 96.03%, 98%, and 96%, respectively, with VGG19 demonstrating the best performance. The study is limited by the use of a single public, slice-based dataset and the absence of external clinical validation; future work will focus on multi-sequence MRI and independent clinical cohorts.

## Introduction

Brain tumors are among the most serious and life-threatening conditions in neurological medicine due to their diverse biological behavior, clinical presentation, and treatment response [1]. Common tumor types include gliomas, meningiomas, and pituitary adenomas, each presenting distinct diagnostic and therapeutic challenges. Gliomas are typically infiltrative and require multimodal treatment strategies, meningiomas may cause neurological symptoms through mass effect despite often being benign, and pituitary tumors can lead to significant endocrine dysfunction [2–4].

Magnetic Resonance Imaging (MRI) is the imaging modality of choice for detecting and evaluating these tumors due to its ability to differentiate between soft tissues like tumor boundaries, edema, and involvement of surrounding structures [5]. Clinical assessment commonly relies on multiple MRI sequences, including T1, T2, T1-weighted contrast-enhanced (T1-CE), and FLAIR, each contributing complementary diagnostic information. MRI therefore plays a central role not only in tumor detection but also in surgical planning, treatment selection, and longitudinal follow-up. Currently, the diagnostic workflow involves image acquisition, radiologist evaluation for tumor presence and subtype, potential referral for additional imaging or biopsy, and multidisciplinary discussion for treatment planning. Key decision points include identifying suspicious lesions, differentiating tumor types, assessing tumor margins and progression, and determining optimal therapeutic strategies. However, conventional MRI interpretation remains largely visual and subjective, with reported inter-reader agreement in brain tumor reporting around 80–82% and substantial variability across features and readers in delineating tumor boundaries, even among experienced radiologists [6]. Moreover, an observational study measuring total interpretation time of brain MRI studies found that neuroradiologists spent a mean of around 11 to 18 min per brain MRI depending on experience level [7].

Artificial intelligence (AI), particularly deep learning, has demonstrated significant promise in automating medical image analysis and supporting clinical decision-making. Beyond diagnostic tasks, AI has been increasingly applied to drug discovery, personalized treatment planning, and clinical risk stratification, offering potential improvements in accuracy, efficiency, and cost-effectiveness [8]. In the context of brain tumor imaging, several deep learning models for MRI-based detection and multi-class classification of gliomas, meningiomas, pituitary tumors, and normal brain tissue have reported high diagnostic performance on benchmark datasets, often achieving accuracies exceeding 95% [9, 10], with recent studies further demonstrating the effectiveness of transfer learning–based architectures such as VGG-16 for multi-class tumor classification and enhanced feature extraction [11]. In parallel, ongoing advancements in deep learning architectures continue to improve robustness and generalization across diverse imaging conditions, further reinforcing the potential of AI-driven tumor analysis [12]. Despite these promising results, many prior works focus primarily on model optimization and performance evaluation, with limited attention to deployment, usability, and integration into clinically oriented workflows.

Recent research and real-world implementations show a growing shift toward web-based AI healthcare systems, where deep learning models are integrated into interactive platforms and clinical dashboards to support decision-making, imaging analysis, and patient monitoring [13, 14, 15]. These web-based AI interfaces can be situated within the current clinical workflow to assist radiologists at the image evaluation and decision-support stage, providing automated tumor classification and explainable outputs that support clinician decision-making. Such systems do not replace radiologists but augment critical decision points, potentially reducing interpretation time, highlighting subtle imaging features, and supporting consistent evaluations across cases. Development of these clinically integrated AI tools aligns with FDA guidance on Good Machine Learning Practice (GMLP), emphasizing robust validation, representative datasets, explainability, and lifecycle management to bridge the gap between research models and usable clinical decision-support systems [16].

As summarized in Table 1, a range of deep learning architectures has been explored for medical image classification.

**Table 1:** Benchmark of different AI models in image classification tasks, including but not limited to brain tumor MRI studies

Model	Task	Strength	Example
Classic CNNs	Image classification	Fast and easy to train and adapt, low computational power needed	Have demonstrated high accuracy in brain tumor classification [17]
ResNet	Image classification and feature extraction	Reducing vanishing gradient problems in deep networks	Tumor identification in radiology and pathology [18]
Vision Transformer (ViT)	Image classification and pattern recognition	Performs well on smaller datasets and handles complex patterns	White blood cell classification; retinal disease diagnosis [19]
YOLO	Real-time object detection (helps with classification tasks)	Offers real-time processing capabilities with high accuracy	Identifying colon polyps or fractures in X-rays [20]
DenseNet	Image classification and feature extraction	Enhances information flow between layers	Cancer diagnosis; diabetic retinopathy detection [21]
VGG16	Image classification	Simple, deep convolutional stack; easy to fine-tune	Distinguishing between healthy and diseased tissue [22]

While models such as ResNet and DenseNet achieve strong performance through deep feature extraction, they often require substantial computational resources and lack transparency, limiting real-time clinical deployment and interpretability. Vision Transformers (ViTs) show potential in capturing complex patterns but typically demand large datasets and specialized hardware. Object-detection frameworks such as YOLO offer real-time capabilities but are less commonly optimized for fine-grained tumor subtype classification. Simpler architectures such as CNNs and VGG-based models are easier to fine-tune and deploy but may require careful optimization to balance performance and generalizability.

Despite the rapid growth of AI research in neuro-oncology, clinical adoption remains limited. Regulatory constraints, the need for external validation [23], and the scarcity of FDA-cleared AI tools specifically targeting brain tumor diagnosis [24] underscore the translational gap between algorithm development and routine clinical use. Although the FDA has cleared hundreds of medical AI algorithms, primarily for general radiology tasks, only a handful are designed for brain tumor analysis, for example, VBrain for tumor auto-contouring, MRIMath i2Contour for glioblastoma segmentation, and NeuroQuant^®^ Brain Tumor for metastatic and meningioma quantification, highlighting the limited number of tools directly applicable to neuro-oncology workflows [25–27].

Even with significant advances in deep learning for brain tumor classification, most existing studies focus primarily on offline model development and evaluation, with limited emphasis on real-time deployment and clinical usability. Furthermore, few works integrate multiple CNN architectures within a unified framework while providing an accessible web-based interface for end users. Additionally, the reproducibility and interpretability of such systems remain underexplored. This study addresses these challenges by developing a scalable, modular web-based platform that integrates multiple deep learning architectures—CNN, VGG19, and Xception—within a single user-oriented interface. Unlike prior systems, such as Aksoy et al. [28], focused primarily on explainability or single-model deployment, the proposed platform enables real-time multi-model comparison and benchmarking without requiring programming expertise. The key contributions of this work include:

- a modular, plug-and-play architecture supporting rapid integration of multiple AI models;
- real-time inference and comparative evaluation within a unified interface; and.
- a clinically oriented workflow designed to support practical deployment and future extensions, such as explainability and segmentation tools.

Collectively, these contributions bridge the gap between algorithm development and practical deployment, offering a reproducible and extensible platform for AI-assisted neuro-oncology.

## Methodology

### Study design and overview

This study follows a quantitative, experimental design focused on developing and validating a modular, web-based artificial intelligence (AI) interface for brain tumor classification from magnetic resonance imaging (MRI). The objective is to evaluate whether deep learning models can be packaged into a clinically usable, web-accessible decision support tool rather than remaining as research code. This design is consistent with recent web-deployed, explainable AI platforms for brain tumor diagnosis that combine deep learning-based classification with user-friendly web interfaces for clinical use [29].

**Fig. 1:**
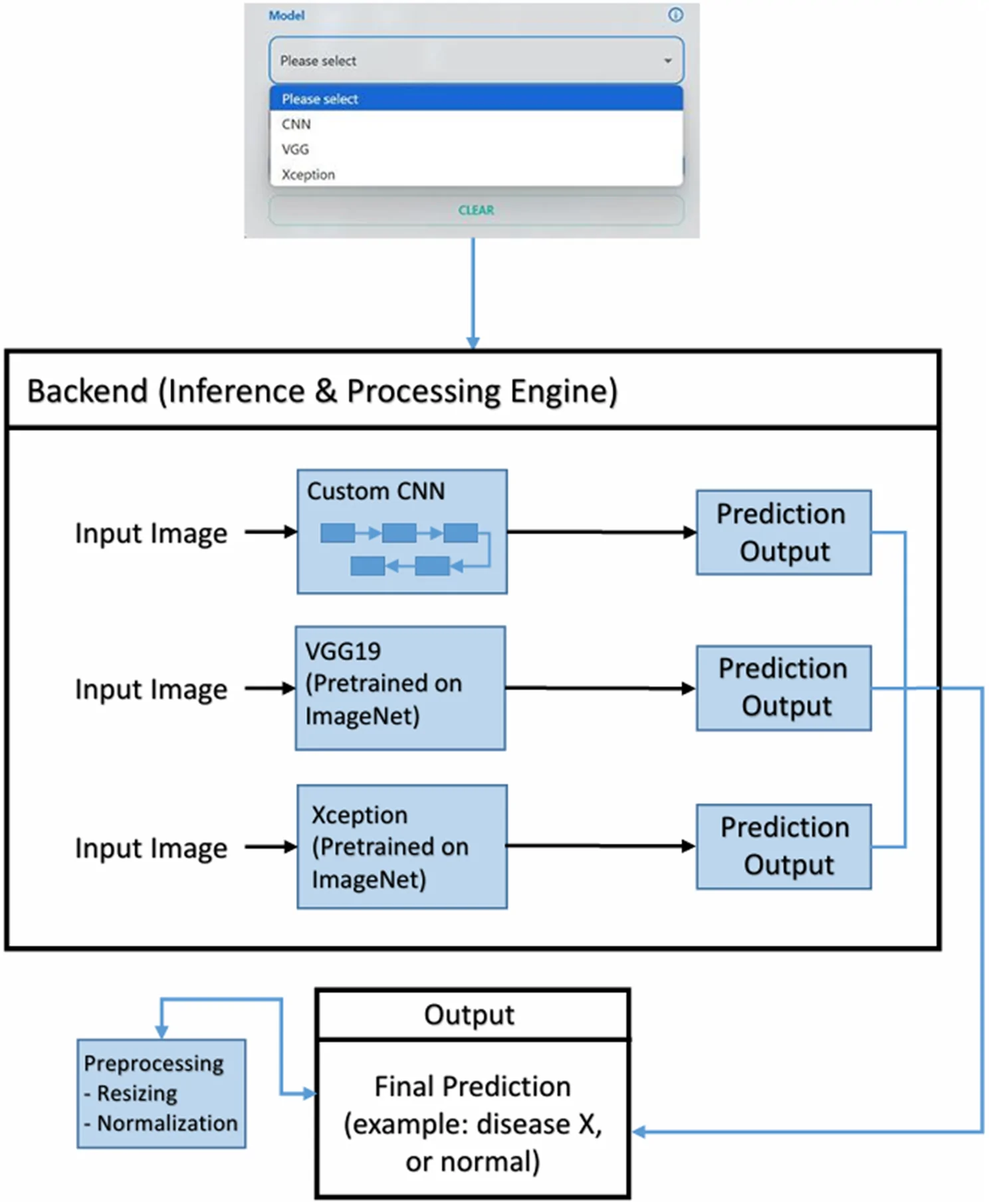
Proposed deep learning workflow

The proposed workflow in Fig. 1 integrates three supervised deep learning classifiers, a custom convolutional neural network (CNN) and two pretrained transfer-learning architectures (VGG19 and Xception); into a single, unified platform that includes both a front end (user interface) and a back end (inference and preprocessing engine).

VGG19 and Xception were selected because they have demonstrated strong performance in medical imaging classification tasks and can leverage transfer learning to improve feature extraction on limited MRI datasets while maintaining computational feasibility for web deployment. For example, studies employing transfer learning approaches using VGG19 have shown high accuracy in brain tumor classification tasks, and Xception frequently outperforms other transfer learning models such as MobileNetV2 and Inception variants due to its efficient depthwise separable convolutions. A custom CNN is included as a baseline to assess the benefit of transfer learning relative to a model trained from scratch [30].

The methodological pipeline comprises five key stages:

1. Data acquisition and preprocessing: collection of labeled brain MRI slices and application of standardized preprocessing steps before training.
2. Model architecture design and training: development of the custom CNN and adaptation of the pretrained VGG19 and Xception models on the multiclass tumor dataset.
3. Model evaluation and performance benchmarking: assessment of accuracy, precision, recall/sensitivity, F1-score, and confusion matrices across tumor classes to compare model behavior.
4. Integration into a modular web interface: deployment of the trained models in a browser-accessible platform that allows non-programmers to upload MRI images and obtain automated predictions.
5. Validation of the interface functionality and scalability: verification that the system performs consistently, responds quickly, and can support additional models in the future (for example, segmentation modules).

**Fig. 2:**
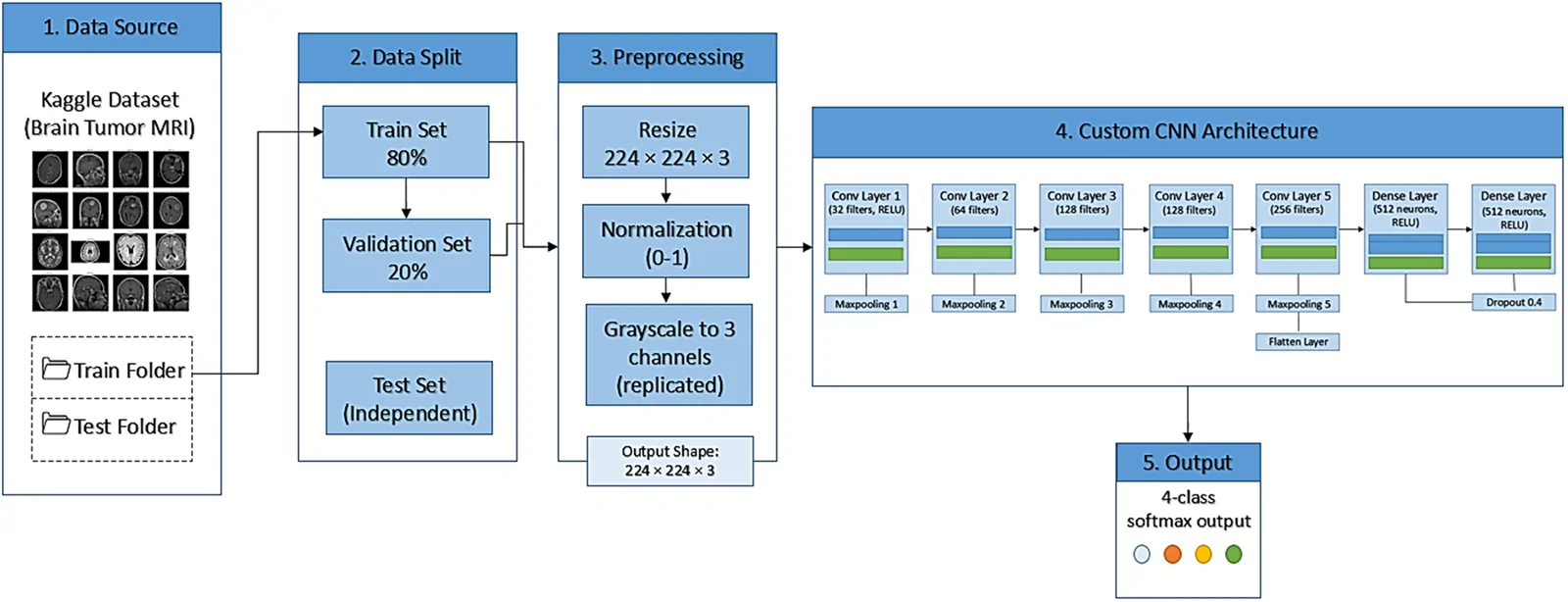
Custom CNN overall workflow diagram

**Fig. 3:**
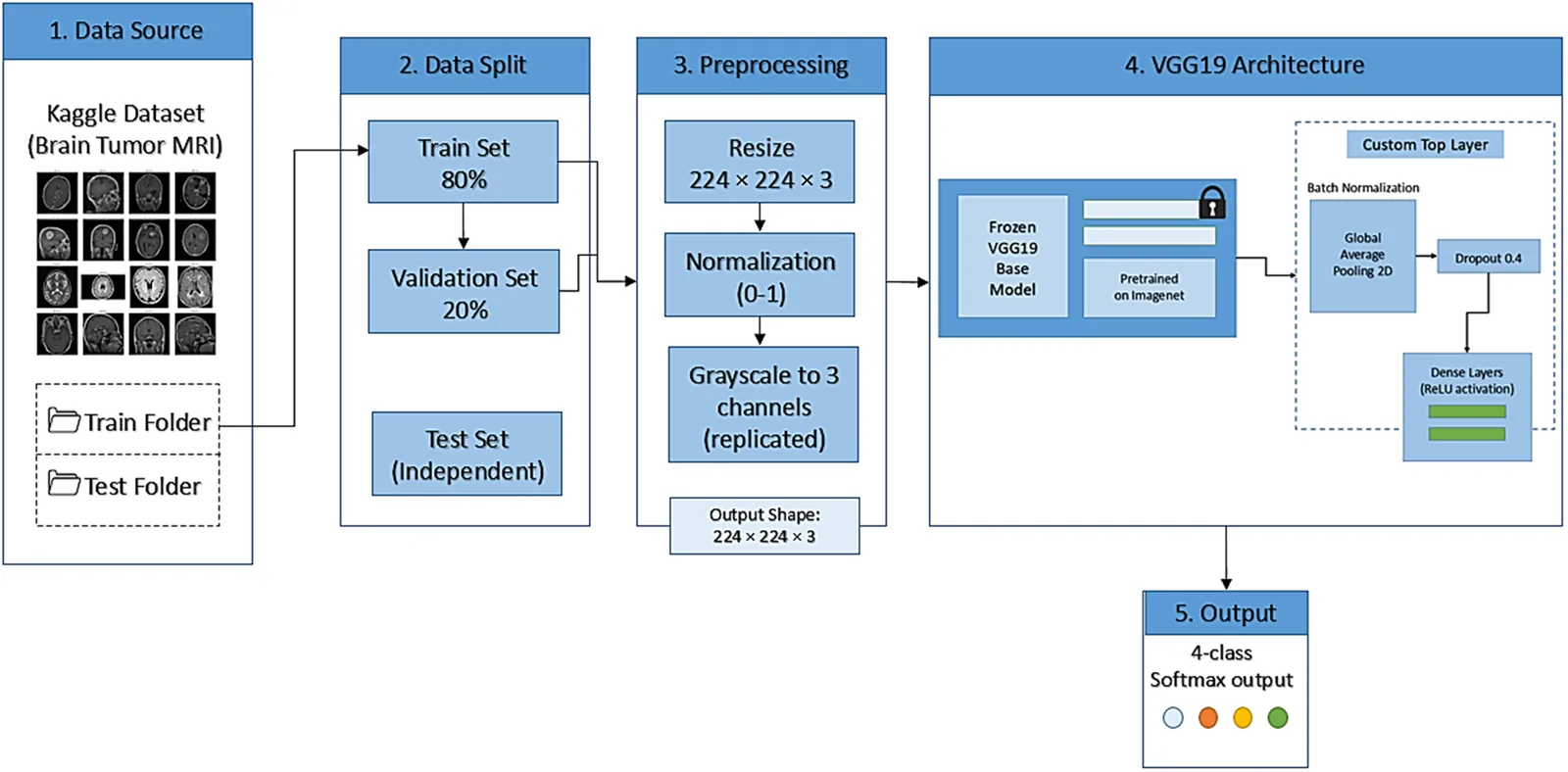
VGG19 overall workflow diagram

**Fig. 4:**
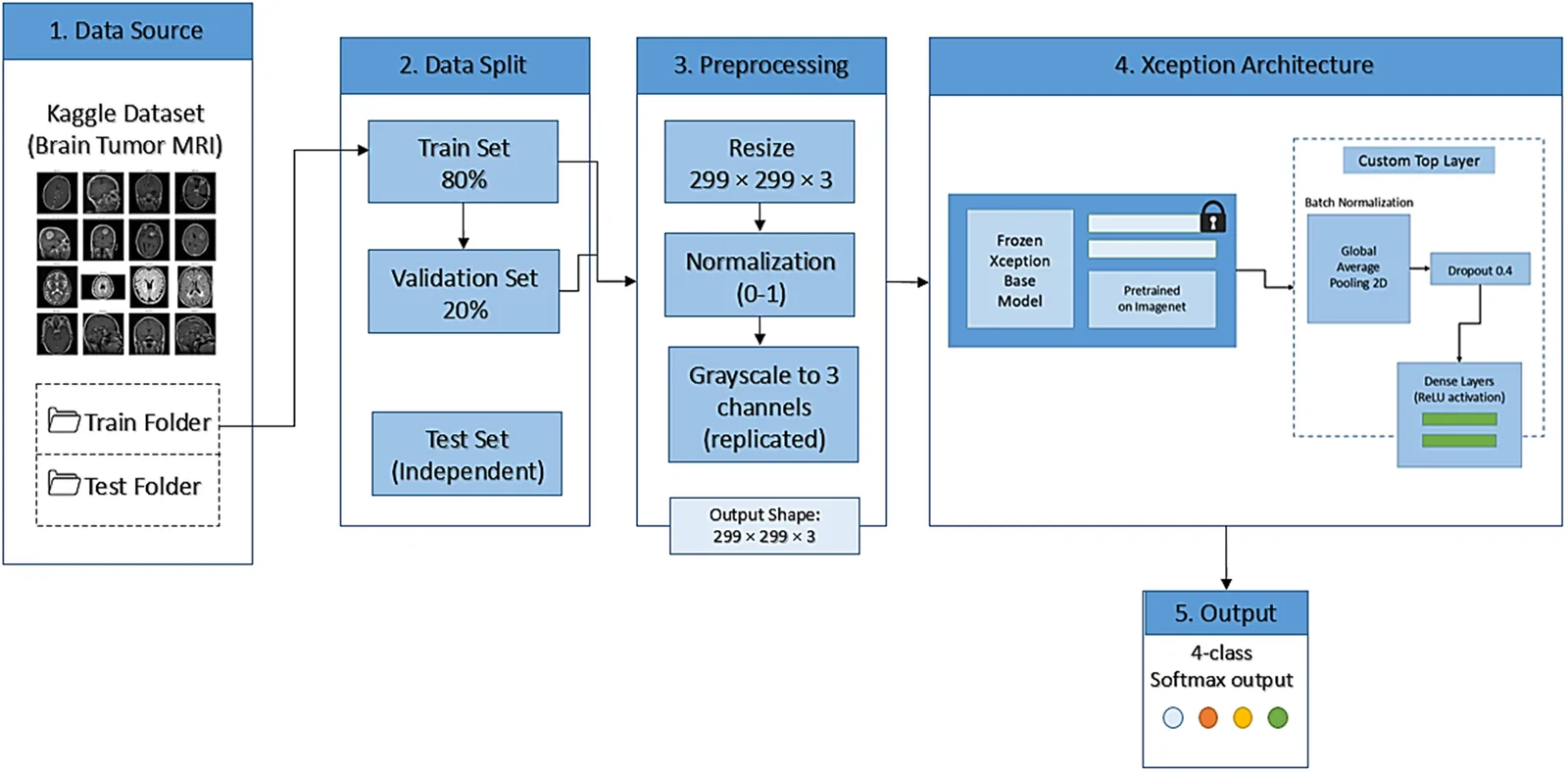
Xception overall workflow diagram

In addition, the overall workflow of the proposed brain MRI classification system is illustrated in Figs. 2, 3 and 4. The diagrams present the complete data flow, including dataset preparation, data splitting, preprocessing operations, and the architectures of the custom CNN, VGG19, and Xception models used for multi-class tumor classification, with an output of one of the 4 classes: glioma, meningioma, pituitary, and no tumor.

### Data description

A publicly available T1-weighted contrast-enhanced brain MRI dataset was obtained from Kaggle (Nickparvar, 2021) [31]. This dataset is widely used in recent brain tumor classification studies and benchmarking work. Several recent deep learning-based MRI classification studies have adopted this Kaggle dataset as a benchmark for multi-class brain tumor classification tasks, confirming its suitability for evaluating CNN and transfer-learning models [32]. The dataset contains 7,023 labeled images, categorized into four diagnostic classes: glioma, meningioma, pituitary tumor, and no tumor. These classes reflect common tumors encountered in clinical neuro-oncology as well as normal brain tissue, which makes the dataset suitable for multi-class classification purposes.

This dataset lacks associated patient demographic information, tumor grade, size, and anatomical location metadata, limiting assessment of performance across clinically relevant subgroups. Slice selection criteria are not described, and it is unclear whether included images represent diagnostically challenging cases or more obvious tumors. As the dataset is single-slice and single-center, model performance may not generalize to whole-volume, multi-sequence MRI scans typically used in clinical practice. Additionally, scanner specifications, imaging protocols, and inter-institutional variability are unknown, introducing potential biases. Future work will include multi-sequence, whole-volume datasets and independent clinical cohorts to improve real-world applicability and reduce dataset-induced biases.

The images were previously divided by the dataset provider into a training subset (*n* = 5,712 images) and a testing subset (*n* = 1,311 images); however, no patient identifiers or metadata are included. Therefore, it is not possible to confirm whether slices in the training and test sets originate from distinct patients. Consequently, a risk of train-test contamination cannot be ruled out, and reported accuracies may be slightly overestimated due to potential overlap. Additionally, the absence of patient-level identifiers prevents verification of full independence between training and test sets. Some slices from the same patient may appear in both sets, potentially inflating performance metrics and limiting generalizability to new patients. These dataset limitations mean that the results should be interpreted as a demonstration of the platform and deployment framework, rather than a claim of clinical performance.

During model development in this study, the training subset was further split internally into training (80%) and validation (20%) to monitor performance and reduce overfitting across epochs.

All images are stored as grayscale JPEG files, which simplifies preprocessing, reduces storage/transfer cost, and allows direct ingestion by common deep learning pipelines without an additional DICOM parsing step. The dataset includes primarily axial slices from contrast-enhanced studies, reflecting typical radiology workflow for brain lesion screening.

The four classes are relatively balanced in size, with each tumor class contributing more than 1,600 images and the “no tumor” class contributing 2,000 images, as shown in Table 2. This near-balance helps reduce bias toward any single diagnosis and supports fair comparison between models when reporting accuracy, precision, recall, and F1-score.

**Table 2:** Dataset composition

Class	Training Images	Testing Images	Total
Glioma	1,321	300	1,621
Meningioma	1,339	306	1,645
Pituitary	1,457	300	1,757
No Tumor	1,595	405	2,000
**Total**	**5**,**712**	**1**,**311**	**7**,**023**

**Fig. 5:**
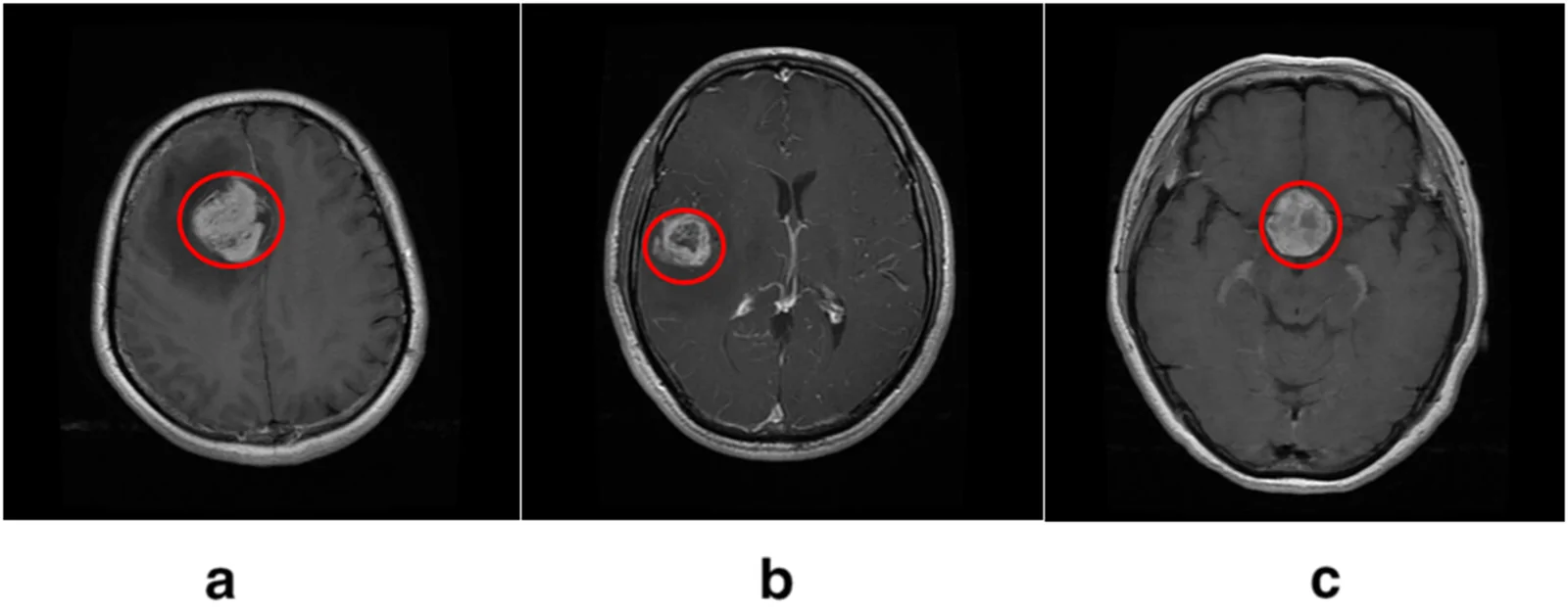
Different brain tumor classes (a. Meningioma, b. Glioma, c. Pituitary)

Each brain tumor class arises from different cell types and regions within the brain, each with distinct characteristics, growth patterns, and clinical implications:

- Meningiomas: Meningeal tumors that are usually benign and slow-growing but can press on nearby brain structures to cause neurological symptoms. (Fig. 5a)
- Gliomas: tumors that originate from the glial cells that support neurons; because they are invasive, they frequently infiltrate neighboring brain tissue and require multimodal treatments. (Fig. 5b)
- Pituitary: hormonal imbalances and a variety of systemic or vision-related symptoms can result from abnormal growths in the pituitary gland. (Fig. 5c)

In summary, this Kaggle dataset, consisting of 7,023 MRI images across four clinically relevant classes, is consistent with datasets used in recent deep learning studies on automated brain tumor classification, and it provides a good benchmark for evaluating the CNN, VGG19, and Xception models, while acknowledging that it may not fully represent the variability of clinical imaging across institutions.

### Data preprocessing

Preprocessing was standardized to ensure comparability across models and reproducibility of results. All input MRI images passed through the same preprocessing steps before being used for training, validation, and testing, so that differences in performance could be attributed to the model architecture rather than to differences in input preparation. These choices are consistent with typical MRI preprocessing pipelines described in recent deep learning reviews, which emphasize standardized resizing, intensity normalization, and (when used) controlled data augmentation to support robust brain tumor classification [33].

- Resizing: Each MRI image was resized to 224 × 224 pixels for the custom CNN and VGG19 models, and to 299 × 299 pixels for the Xception model, in order to match the required input dimensions of each model’s first layer. This ensured that all models received standardized inputs during inference in the web interface.
- Normalization: Pixel intensity values were rescaled to the range [0, 1] by dividing raw pixel values by 255 prior to training. This normalization step helps stabilize gradient updates and improves convergence during optimization.
- Color mode: All images were originally treated as grayscale to match the format of the dataset. But, since pretrained models like Xception and VGG19 require a three-channel input, each one of the grayscale images ended up being replicated across all three channels (from 1×H×W to 3×H×W). This keeps preprocessing straightforward and uniform across all models, maintains the original grayscale information, and satisfies the input requirements of the pretrained architectures.

Before training, each image and its label were checked for consistency to reduce the risk of label noise, which can negatively affect supervised learning.

### Data augmentation

To improve model generalization and reduce overfitting, data augmentation was applied during training. These transformations simulate realistic variations commonly observed in clinical imaging, such as changes in orientation, scale, illumination, and position. Specifically, random rotations, horizontal shifts, vertical shifts, zooming, brightness adjustment, and horizontal flipping were used. Data augmentation increases the effective size and diversity of the training dataset without altering the underlying class distribution, allowing the models to become more robust to variability in MRI acquisition conditions while preserving the diagnostic content of the images.

During training, input images were first preprocessed using the model-specific preprocessing function. Random rotations up to 15°, horizontal and vertical translations of up to 10% of the image dimensions, zoom variations up to 15%, and brightness adjustments within the range of 0.85–1.15 were applied. In addition, horizontal flipping was enabled, and newly created pixels were filled using the nearest-neighbor strategy.

### Hyperparameter optimization

Hyperparameters were fixed across all experiments and selected based on prior literature and preliminary testing. Key parameters, such as learning rate and dropout rate, were chosen within commonly used ranges and exhibited stable performance during initial experiments, although no formal sensitivity analysis was conducted. Systematic hyperparameter optimization and sensitivity analysis will be considered in future work.

### System architecture

The proposed platform is built as a modular, two-tier system consisting of a client-facing frontend and a model-serving backend. This separation allows the system to be used by non-technical users while keeping the deep learning models, preprocessing logic, and inference pipeline consistent and centrally controlled.

#### Frontend (user interface layer)

The frontend is implemented using React. It provides a graphical interface where the user can:

- Upload a brain MRI scan,
- Select the desired model (custom CNN, VGG19, or Xception),
- Trigger preprocessing and inference,
- View the predicted tumor class.

The interface is designed to require no programming skills. The user experience is kept minimal: image upload, model selection, and classification output. The frontend also performs basic validation (e.g., allowed file type and size) before sending the image to the backend.

In addition to the classification result, the interface can display model confidence scores so that the user can assess how strongly the selected model supports its prediction. The design is modular, which implies that new output elements (for example, visual explanation maps in future work) can be added to the same view without changing the core workflow.

#### Backend (inference and processing layer)

The backend is implemented using FastAPI. It is responsible for receiving image data from the frontend, applying preprocessing, loading the selected trained model, and returning the predicted class as output.

The backend exposes dedicated endpoints for:

1. Image preprocessing,
2. Model inference,
3. Model selection and configuration.

All preprocessing steps (resizing, grayscale handling, and normalization) are performed server-side to ensure that inputs to the models are standardized and reproducible.

The deep learning models are loaded as serialized weights and architectures. The server keeps these models in memory to avoid reloading them for each request, which improves response time during repeated inference. If needed, the backend can be extended to include additional architectures such as segmentation models or attention-based or transformer-based classifiers, without requiring changes to the frontend logic.

#### Model manager / modular design

A central component in the backend is the model manager. This module:

- Registers each available model (CNN, VGG19, Xception),
- Enforces the correct input shape and preprocessing routine for that model,
- Routes incoming inference requests to the selected model.

By abstracting the models behind a single interface, the system supports “plug-and-play” expansion. A newly trained model can be integrated by adding it to the registry with its input specifications and output classes, without rewriting the rest of the application.

#### Data flow

The overall data flow is as follows:

1. The user uploads an MRI scan and selects a model in the React interface.
2. The image and model choice are sent to the FastAPI backend.
3. The backend applies standardized preprocessing.
4. The appropriate trained model is called by the model manager.
5. The model outputs a predicted class (glioma, meningioma, pituitary tumor, or no tumor), along with optional confidence values.
6. The result is returned to the frontend for display.

### Deep learning architecture

Three different deep learning models were designed and integrated into the platform. The first model was a custom-built CNN model, while the other two, VGG and Xception, were pretrained models. These models were chosen to offer a fair comparison between two highly effective transfer learning models that are well-known for their efficacy in image classification tasks and a lightweight, task-specific CNN architecture.

#### Custom CNN model

The first model was a basic Convolution Neural Network that was built from scratch. Given its effectiveness in tasks like image classification [34], object detection and medical image analysis, this model will serve as a good foundation to evaluate the ability of a simple deep learning model to differentiate between and classify images of tumors and those without, while also specifying what type of tumor it may be.

Another reason why CNNs are good at interpreting medical images is because they use convolutional layers which help them detect edges in early layers and more complex patterns later on in the deeper layers [35].

Overall, starting with this simple CNN model provided a clear view of how well a lightweight model will perform when trained on medical images, specifically brain MRI scans, in comparison to more complex pretrained models.

##### Model architecture

**Fig. 6:**
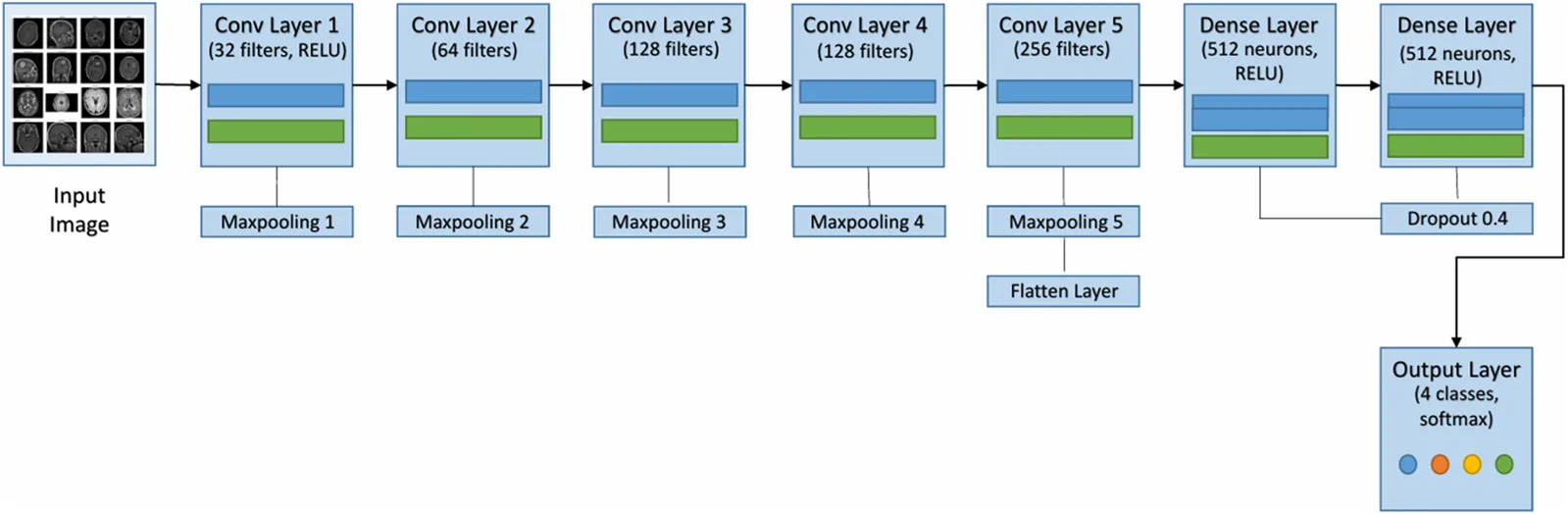
Custom CNN model architecture

For the network to be able to capture a variety of features, from simple edges to more complex structures in the images, five convolutional layers with increasing neuron sizes were stacked illustrated in Fig. 6. Such an approach is rather effective when dealing with MRI images because they are made up of intricate patterns that need to be taken into consideration during training.

In order to decrease the spatial dimensions of the feature maps, max-pooling layers were added after each convolutional layer, bringing the total to five layers as well. This structure will in turn aid in lowering the complexity of computation and decreasing the number of parameters to avoid overfitting. In addition, it will also introduce spatial invariance which makes sure the object, being the tumor in this case, is detected wherever its position in the image.

In order to prepare the data for the fully connected layers, a flatten layer is added, as it transforms the 2D feature maps into a 1D vector. This step will allow for the integration of the spatially extracted features into a format appropriate for classification tasks. Thanks to the added dense layer with 512 neurons, the model will be able to learn intricate combinations of the features that the convolutional layers have extracted.

To further help the network recognize the intricate patterns in the MRI images, the ReLU activation function is applied on the hidden layers for non-linearity. Some of its main advantages include reducing the vanishing gradient issue and being computationally efficient. In addition, another tool, softmax, was also applied, specifically to the output layer in order to facilitate multi-class classification tasks by transforming the final outputs into probability distributions across the target classes (glioma, meningioma, pituitary and no tumor).

##### Model parameters

To successfully train the CNN model, avoid overfitting, and balance the learning performance, a set of parameters were chosen.

The model was trained for a maximum of 30 epochs, which is considered a moderate range frequently used in image classification tasks. The images were already divided into two folders, training and testing, so, in addition to that, the first folder was split into 80% for training and 20% for validation. A batch size of 32 was chosen since it provides a good balance between memory usage and the speed of the model convergence.

To avoid overfitting as much as possible a dropout rate of 0.4 was applied to the dense layer, which means that a random 40% of the neurons is deactivated during training. That way, the model is forced to learn more robust features so it can make better predictions on new and unseen data. These choices were guided by empirical experimentation on the validation subset to balance training stability, computational cost, and the risk of overfitting.

Given that we’re working with medical images which have features that may be more difficult to read, the Adam optimizer was selected because of its adaptive learning rate and capacity to converge more quickly. Mathematically speaking, the Adam (Adaptive Moment Estimation) optimizer updates each parameter 𝜃 using both the first moment or mean (Eq. 1) and second moment or variance (Eq. 2) of the gradients:

1 $$\:{m}_{t}\mathbf{}={{\upbeta\:}}_{1}\mathbf{}{m}_{t-1}\mathbf{}+\left(1-{{\upbeta\:}}_{1}\mathbf{}\mathbf{}\right){g}_{t}$$

2 $$\:{v}_{t}\mathbf{}={{\upbeta\:}}_{2}\mathbf{}\mathbf{}{v}_{t-1}\mathbf{}+(1-{{\upbeta\:}}_{2}\mathbf{}){g}_{t}^{2}$$

3 $$\:{{\uptheta\:}\:}_{t+1}=\:{{\uptheta\:}\:}_{t}-\:\alpha\:\:\frac{{m}_{t}/(1-{{\upbeta\:}}_{1}^{t})}{\sqrt{{v}_{t}/(1-{{\upbeta\:}}_{2}^{t})}+\epsilon}$$

where $\:{g}_{t}$ is the gradient at time step t, and $\:{{\upbeta\:}}_{1},{{\upbeta\:}}_{2}$, are decay rates.

The parameters are then updated as shown in Eq. 3:

4 $$\:L=\sum_{n=1}^{C}{y}_{n}\mathrm{l}\mathrm{o}\mathrm{g}\left({p}_{n}\right)$$

with $\:\alpha\:$ being the learning rate and $\:\epsilon$ a small constant to avoid division by zero.

This allows Adam to adaptively adjust the learning rate for each parameter, improving convergence speed and stability.

In addition, since the model is intended for multi-class classification, the categorical crossentropy loss function, which calculates the difference between the expected and actual class probabilities, was employed.

Mathematically, for * C* classes, the categorical crossentropy loss function is defined as shown in Eq. 4:

Where $\:{y}_{n}\:$is the true label (1 for the correct class and 0 for the others), and $\:{p}_{n}$ is the predicted probability for class n.

This loss function penalizes predictions that deviate from the true class, guiding the model to give higher probabilities to the correct categories which in turn improves classification accuracy [36].

As for the computational time and resources, the whole training process along all 30 epochs took around fifteen minutes and was done using NVIDIA T4 GPU provided via Google Colab Pro.

#### VGG19 model

The second model used in this project is one with a VGG19 base, a CNN architecture developed by the University of Oxford’s Visual Geometry Group (VGG) [37]. VGG19 is a more complex version of the VGG family, consisting of 19 layers, 16 of which are convolutional layers and 3 fully connected layers. It is mostly well-known for its 3 × 3 convolution filters which are stacked on top of each other. From detecting edges and textures in the early layers, to distinguishing more abstract, high-level representations in the deeper layers, this architecture allows the network to capture a range of important features.

This model was already pretrained on Imagenet, which is a large-scale database with over 14 million labeled images spanning 1,000 object categories, mainly used for visual object recognition. Using its pretrained weights on ImageNet, VGG19 was used to classify, leveraging the features it extracts for classification purposes.

Considering that medical images, specifically MRI scans, contain minute details, the deep and homogeneous structure of VGG19 is especially advantageous and is a good choice for categorizing diseases like glioma, meningioma, pituitary tumors, and healthy brains because of its capacity to learn and generalize these complex visual patterns.

##### Model architecture

**Fig. 7:**
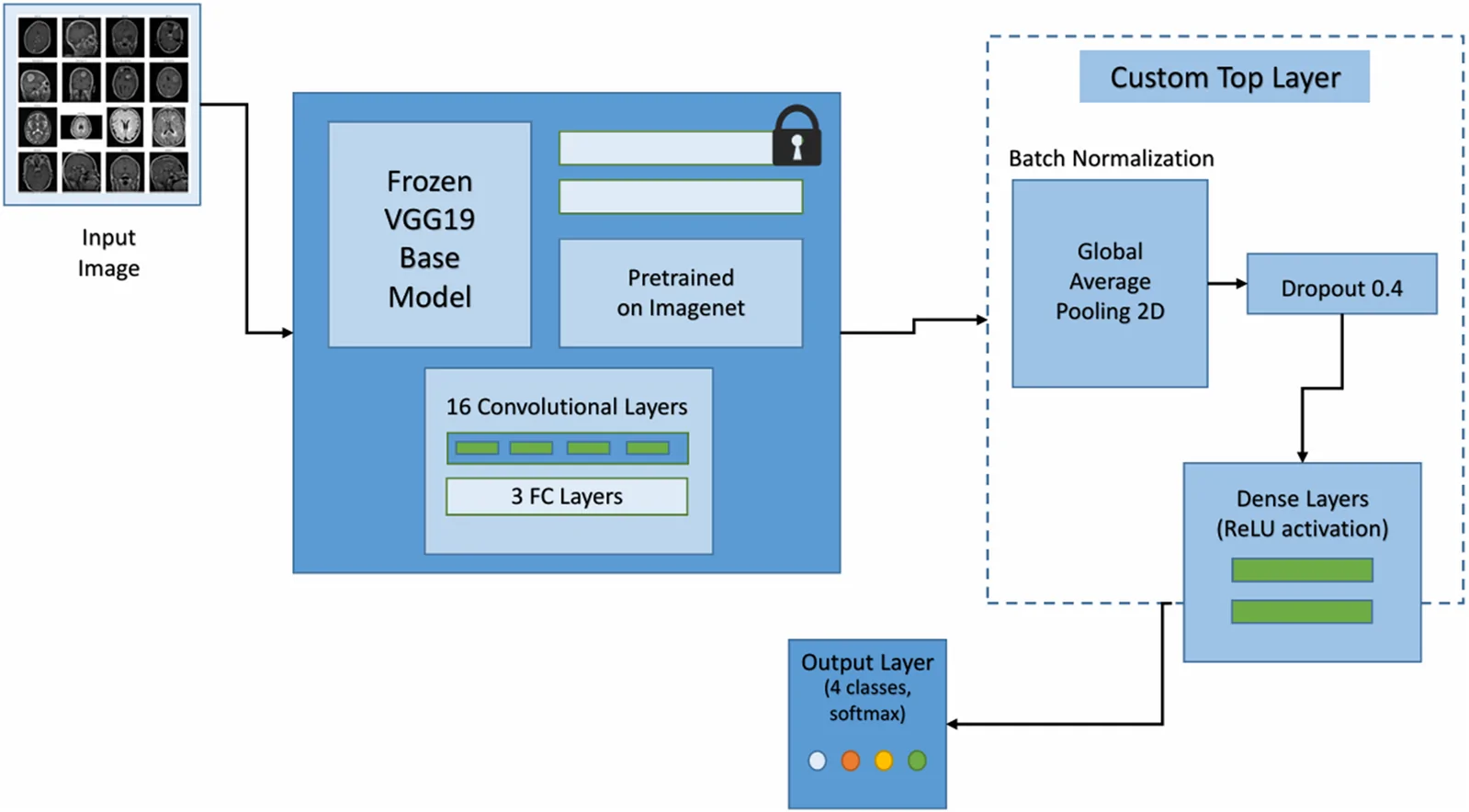
VGG19 model architecture

As previously mentioned, the original architecture of this model consisted of a total of 19 base layers illustrated in Fig. 7. Given that the dataset is generally small, and in order to avoid overfitting, the VGG19 layers were frozen. This also helps decrease the number of trainable parameters, in turn making the whole training process faster and less resource-intensive, especially when it comes to the computing power. That way it was used as more of a fixed feature extractor. A custom top layer to be added on top of the frozen VGG19 model was built, which will learn to associate the extracted features with the dataset’s class labels.

The model makes use of GlobalAveragePooling2D. That way, instead of flattening these large feature maps directly, it creates a feature vector by averaging each feature map into a single value. To simply put it, GlobalAveragePooling2D reduces the spatial dimensions (width and height) of each feature map to a single number by calculating the average of all values in each feature map. This preserves the most crucial global spatial information while drastically lowering the number of trainable parameters, improving the model’s generalization and lowering the chance of overfitting [38].

Two fully connected (dense) layers are then added in order to use the extracted features to learn intricate patterns. The output probabilities are then generated by a dense layer and a softmax activation function, which makes it appropriate for multi-class classification tasks like identifying various kinds of brain tumors in our case. In addition, batch normalization was applied in order to improve the speed, stability and performance of the network.

##### Model parameters

This model was trained for a total of 30 epochs with a batch size of 32. Given that the images were already divided into two folders, training and testing, the first folder was additionally split into 80% for training and 20% for validation.

The Adam optimizer was chosen with a learning rate of 0.001 to guarantee stable learning. To avoid overfitting as much as possible, a dropout rate of 0.4 was applied, which means that a random 40% of the neurons is deactivated during training.

As for the computational time and resources, the whole training process along all 30 epochs took around twenty-five minutes and was done using NVIDIA T4 GPU provided via Google Colab Pro. Collectively, these hyperparameters were tuned to leverage the representational strength of the pretrained VGG19 backbone while keeping training time, memory usage, and overfitting risk acceptable for integration into the modular web platform.

#### Xception model

The project’s third model is based on the Xception architecture, which stands for “Extreme Inception.” It was originally developed in 2017 by Francois Chollet at Google [39].

**Fig. 8:**
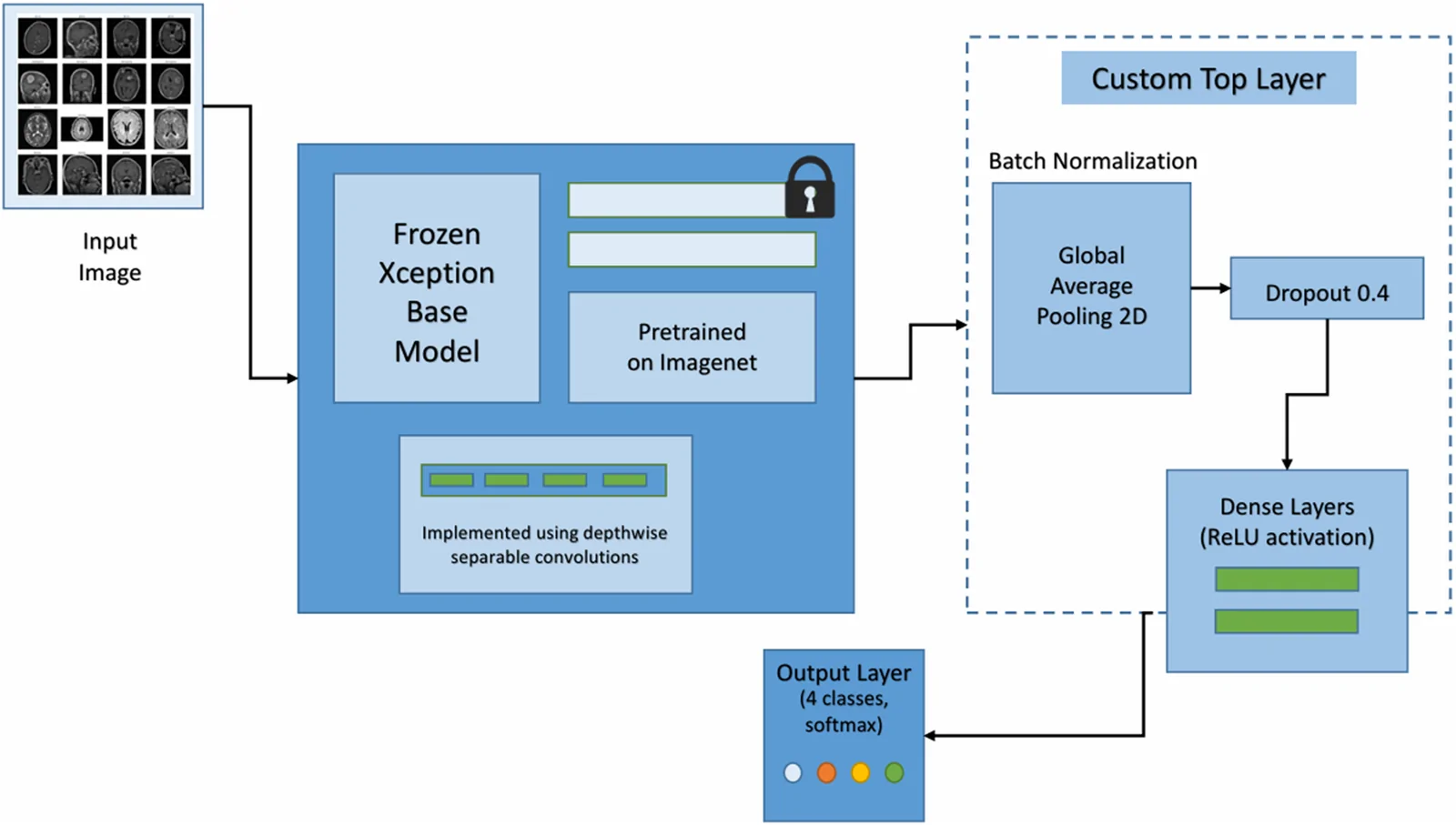
Xception model architecture

By fully replacing the standard Inception modules with depthwise separable convolutions, Xception takes the ideas within the Inception family of models one step further. That way, the convolution process is divided into two stages illustrated in Fig. 8: a pointwise convolution that combines the outputs using 1 × 1 convolutions and a depthwise convolution that applies a single filter to each input channel. As a result, we get a model that is both more efficient and more powerful in terms of feature extraction.

This model was also pretrained on Imagenet, giving it a strong foundation when it comes to identifying complex visual patterns. For this project, its pretrained weights were used as a feature extractor to draw out intricate details in the MRI scans and in turn help classify brain tumors.

##### Model architecture

For this model, a similar method to the VGG19 was applied, which consisted of freezing the Xception base layers in order to decrease computing time and resources.

A custom top layer to be added on top of the frozen Xception model was also built in this case. It included similar layers to the previous VGG19 model. GlobalAveragePooling2D was also utilized to average feature maps and compresses them into a vector. A dense layer with the ReLU activation function was added to learn higher-level representations, while two dropout layers, each with a 40% rate, were added to lessen overfitting. Lastly, class probabilities are predicted by a dense output layer with four units and the softmax activation function. In addition, batch normalization was applied in order to improve the speed, stability and performance of the network.

##### Model parameters

This model was trained for 10 epochs with a batch size of 32. The training folder in the dataset was split into 80% for training and 20% for validation. The Adam optimizer was selected with a learning rate of 0.001 to guarantee stable learning. To avoid overfitting as much as possible, a dropout rate of 0.4 was applied, which means that a random 40% of the neurons is deactivated during training.

As for the computational time and resources, the whole training process along all 10 epochs took around thirty-five minutes and was done using NVIDIA T4 GPU provided via Google Colab Pro. In this configuration, the reduced number of trainable layers and epochs was deliberately chosen to control computational cost while still benefiting from the depth and representational capacity of the Xception architecture.

### Evaluation metrics

A set of common evaluation metrics was used to thoroughly evaluate the classification models’ dependability and performance. These metrics were selected in order to measure not only overall accuracy but also the models’ capacity to manage class imbalances, accurately identify each tumor class, and reduce false positives and false negatives.

Accuracy measures how correct the model is overall by dividing the number of accurate predictions by the total number of predictions (Eq. 5)

5 $$\:Accuracy=\frac{TP+TN}{TP+TN+FP+FN}=\frac{Number\:of\:Correct\:Predictions}{Total\:Number\:of\:Predictions}$$

Precision shows how many of the positive predictions were actually positive In other words, it predicts the model’s ability to avoid false positives (Eq. 6)

6 $$\:Precision=\:\frac{TP}{TP+FP}$$

Recall (Sensitivity) checks how many of the positive predictions were correctly identified. In other words, it helps detect true positives (Eq. 7)

7 $$\:Recall=\:\frac{TP}{TP+FN}$$

F1-Score provides a balance between the mean of precision and recall when classes are unbalanced (Eq. 8)

8 $$\:F1-Score=2\times\:\:\frac{Precision\:\times\:Recall}{Precision+Recall}$$

**Fig. 9:**
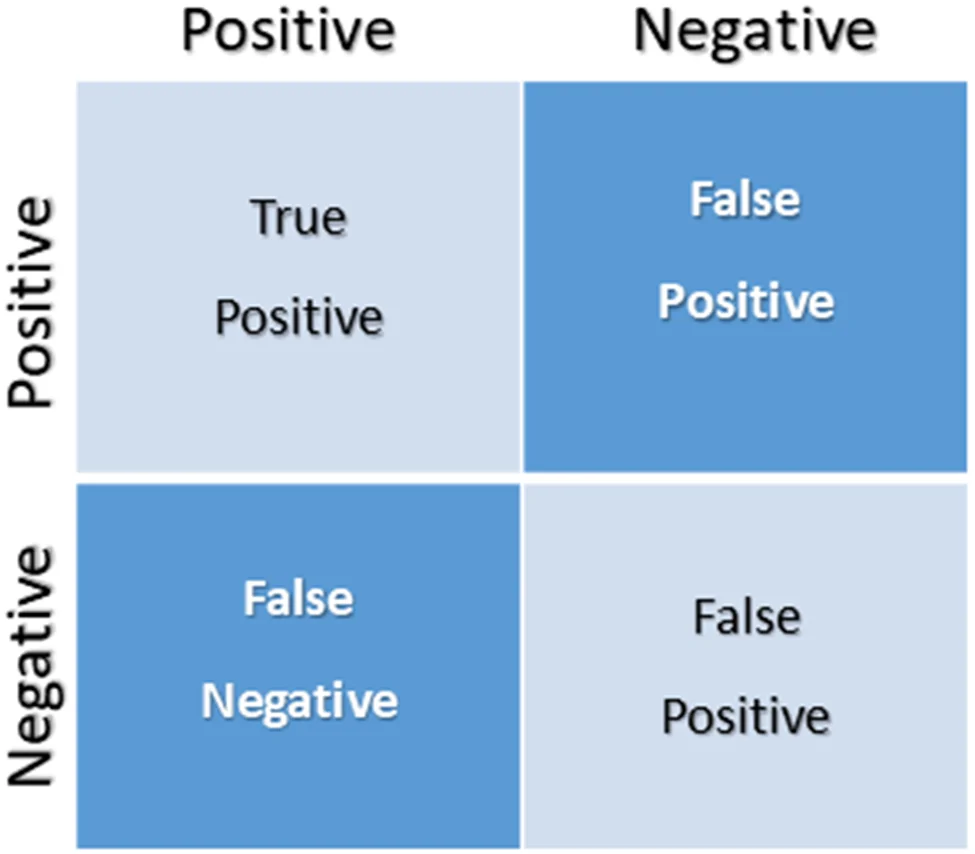
Confusion matrix

Confusion Matrix is a table illustrated in Fig. 9 that displays the number of true positives, true negatives, false positives and false negatives. It helps identifying misclassified images across all classes

- **True Positives (TP)**: The number of cases that were correctly classified as being in the positive class (for example, tumor present when tumor is actually present).
- **True Negatives (TN)**: The number of cases that were correctly classified as being in the negative class (for example, no tumor present when no tumor is actually present).
- **False Positives (FP)**: The number of cases that were incorrectly classified as being in the positive class (for example, tumor present when no tumor is present). This is known as Type I Error.
- **False Negatives (FN)**: The number of cases that were incorrectly classified as being in the negative class (for example, no tumor present when tumor is present). This is known as Type II Error.

Positive Predictive Value (PPV) is the proportion of predicted positive cases that are truly positive (Eq. 9)

9 $$\:PPV=\:\frac{TP}{TP+FP}\:$$

Negative Predictive Value (NPV) is the proportion of predicted negative cases that are truly negative (Eq. 10)

10 $$\:NPV=\:\frac{TN}{TN+FN}\:\:$$

False Negative Rate (FNR) is the proportion of actual positive cases that were incorrectly predicted as negative (Eq. 11)

11 $$\:FNR=\:\frac{FN}{TP+FN}\:\left(1-Sensitivity\right)\:\:$$

Every one of these metrics will help determine just how well the model is being able to accurately classify brain tumor MRI images, ensuring a thorough analysis that goes beyond just the overall accuracy.

### Explainability

While the current study focuses on classification performance and robustness, model interpretability remains a critical requirement for clinical deployment. Regulatory frameworks and best-practice guidelines for medical AI, such as the FDA’s Good Machine Learning Practice (GMLP), emphasize the importance of transparency and explainability to support clinical trust, safety, and accountability in decision-making systems [40].

In future work, we will integrate gradient-based explainability techniques, specifically Grad-CAM, to visualize class-discriminative regions influencing model predictions. These saliency maps will be systematically evaluated through a clinician-in-the-loop study, where radiologists will compare AI-generated heatmaps against expert manual tumor annotations. This evaluation will assess both localization accuracy and clinical relevance, providing quantitative and qualitative validation of model interpretability.

Incorporating explainability analyses alongside expert evaluation will be a key step toward regulatory alignment and clinical translation of the proposed system.

### Computational environment

Training and evaluation were conducted on an NVIDIA T4 GPU (16 GB VRAM) provided via Google Colab Pro, with Tensorflow version 2.19. Code development and debugging were performed on a local machine (Intel Core i7-1065G7, 16 GB RAM) using Python 3.11.4 and the following libraries:

● TensorFlow/Keras for model construction and training,

● OpenCV and NumPy for image preprocessing,

● Matplotlib and Seaborn for visualization,

● Scikit-learn for computation of evaluation metrics.

### Validation and stability

To assess the reliability and stability of the results, a repeated hold-out validation strategy was applied to the custom CNN, which served as the primary baseline architecture in this study. The CNN was selected for repeated evaluation because it was trained from scratch and had substantially lower computational cost compared to the deeper transfer-learning models. In contrast, the transfer-learning models VGG19 and Xception require significantly longer training time and higher computational resources due to their depth and large parameter counts. Therefore, these models were evaluated using a single fixed train–validation–test split to ensure feasibility while maintaining consistent data partitions for fair comparison.

For the repeated hold-out evaluation, the dataset was first combined and randomly split into 80% training, 10% validation, and 10% testing sets. This procedure was repeated over 10 independent runs, with the CNN trained from scratch each time using different random initializations. The model achieved an average accuracy of 96.5% ± 0.53%, demonstrating stable performance across multiple randomized data splits. This approach captures performance variability while preserving strict separation between training, validation, and testing data.

### Methodological summary

The proposed methodology thus ensures a comprehensive and reproducible framework encompassing data preparation, model optimization, statistical evaluation, and system deployment. The hybrid approach combining deep learning with an accessible web interface, provides an adaptable foundation for future clinical translation and integration of AI-based decision support in neuroimaging workflows.

## Results and discussion

### Models’ performance overview without augmentation

The three models Custom CNN, VGG19, and Xception were trained, validated, and tested on the Kaggle brain-tumor MRI dataset (7,023 images).

Table 3 summarizes their overall quantitative performance using accuracy, precision, recall, and F1-score as evaluation metrics.

**Table 3:** Quantitative models’ performance overview without augmentation

Evaluation Metrics	Custom CNN	VGG19	Xception
Train Accuracy (%)	99.73	94.46	94.8
Validation Accuracy (%)	95.02	94.88	95.54
Test Accuracy (%)	96.03	98.00	96.00
Precision (%)	96.00	98.00	96.00
Recall (%)	96.00	98.00	95.00
F1-Score (%)	96.00	98.00	95.00
Epochs	30	30	10
Time (min)	15	25	35
Mean Inference Time (s/image)	0.012	0.3430	0.199

Based on the above results, we can conclude that the VGG19 model appears to be the top performing model in terms of classification, with its 98% test accuracy, as well as high precision, recall and f1-score (98%), which indicates that it effectively balances false positives and false negatives. Even though the CNN model has the best training accuracy, it is less dependable than VGG19 for real-world deployment due to a small drop in test accuracy. Although Xception is quick to train, it performs lower on important metrics. This can further be examined when comparing the confusion matrices of each model, CNN, Xception, and VGG19 respectively, as shown in Figs. 7, 8 and 9.

- CNN: Displayed strong discrimination for “no-tumor” and “pituitary” cases but higher confusion between glioma and meningioma classes, likely due to their overlapping structural characteristics in T1-weighted MRIs.
- Xception: Correctly identified “pituitary” and “no-tumor” cases but showed occasional misclassification between glioma and meningioma, consistent with its lower recall.
- VGG19: Exhibited a highly diagonal confusion matrix, indicating robust classification across all four tumor types with minimal false negatives.

Quantitatively, VGG19 reached class-specific recalls above 97% in all categories, confirming strong generalization.

**Fig. 10:**
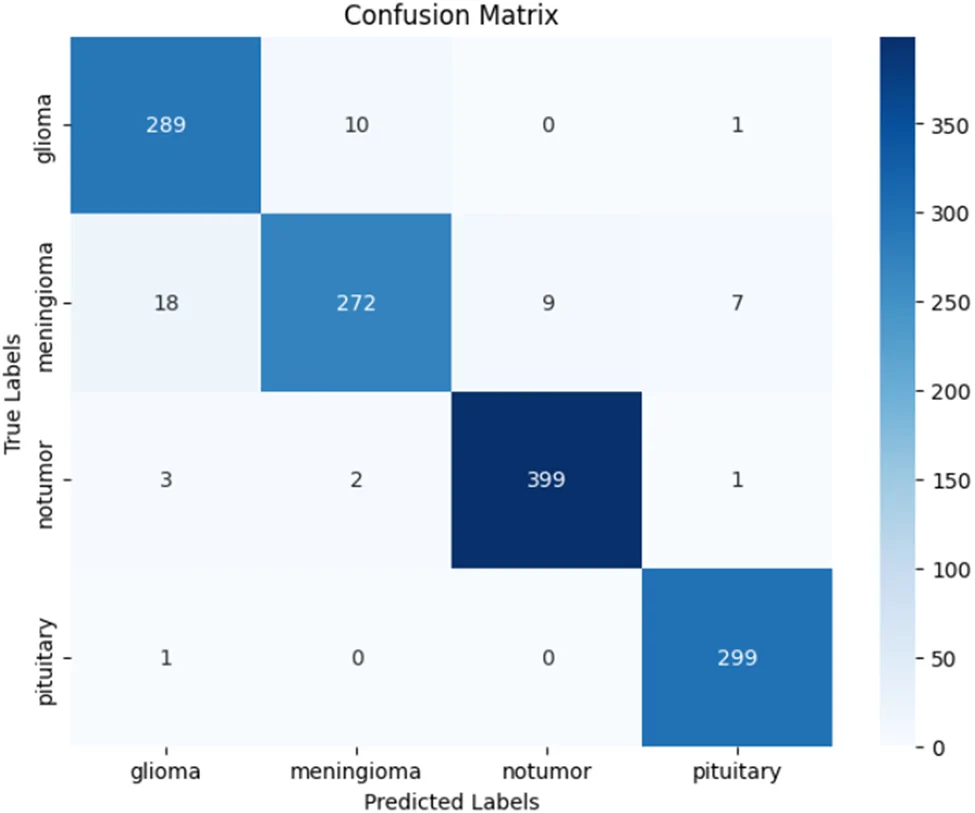
CNN results as a confusion matrix

**Fig. 11:**
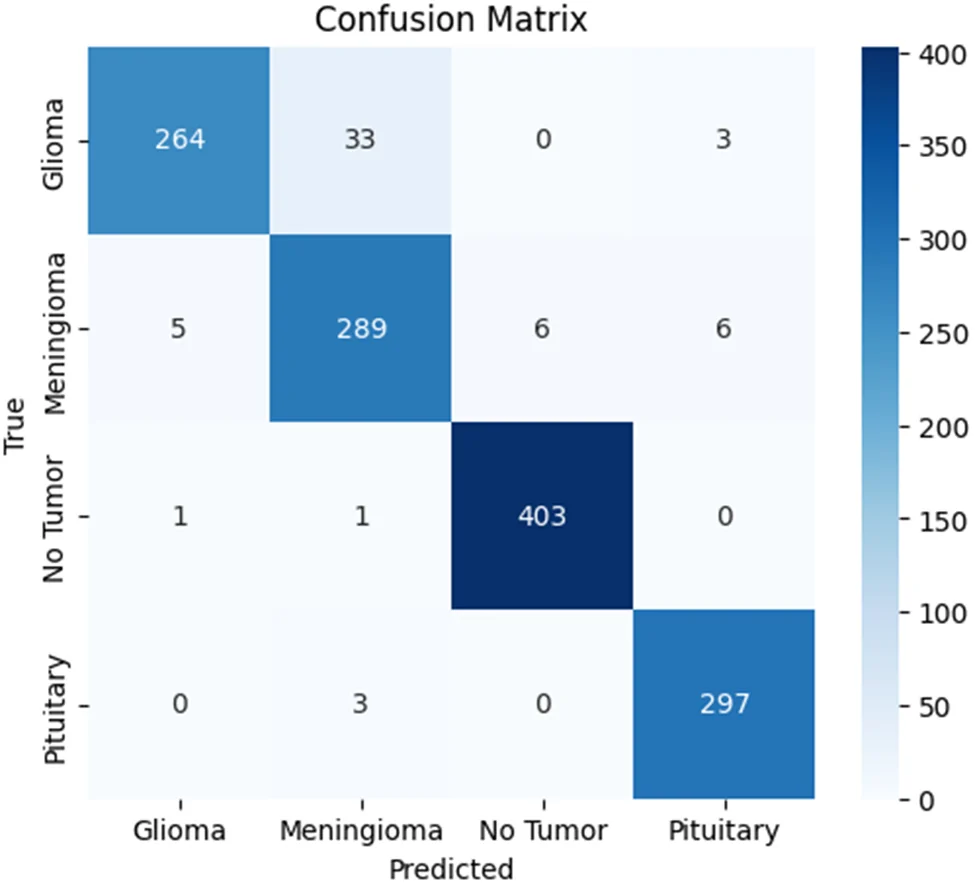
Xception results as a confusion matrix

**Fig. 12:**
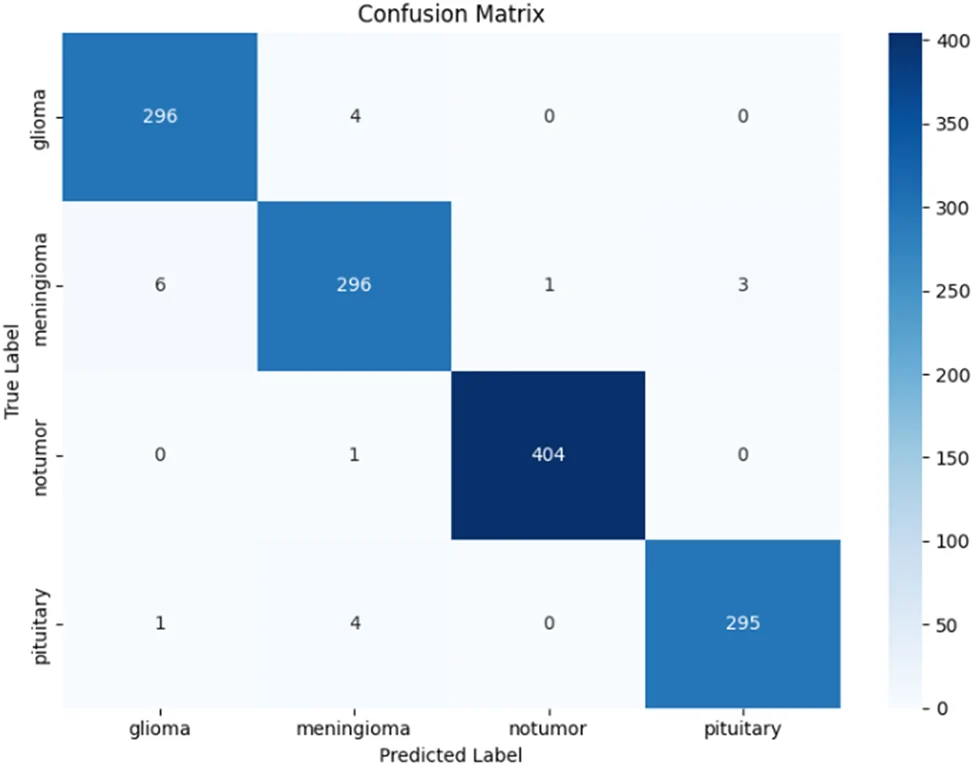
VGG19 results as a confusion matrix

The CNN confusion matrix in Fig. 10 demonstrates strong diagonal dominance, indicating that the majority of samples across all four classes were correctly classified. Specifically, 289 samples were correctly classified as glioma, 272 as meningioma, 399 as no tumor, and 299 as pituitary. A closer inspection of the off-diagonal elements reveals that misclassifications are not uniformly distributed but are concentrated between specific class pairs. In particular, 10 samples from the glioma class were misclassified as meningioma, while 18 meningioma samples were incorrectly predicted as glioma, resulting in a total of 28 glioma–meningioma confusion errors. In the no tumor class, 3 samples were misclassified as glioma, 2 as meningioma, and 1 as pituitary. Finally, for the pituitary class, only 1 sample was misclassified as glioma. These numbers highlight systematic error patterns, showing that glioma and meningioma are the classes most prone to confusion in the CNN model. Overall, the confusion matrix provides valuable insight into model behavior beyond overall accuracy by identifying these class-specific trends.

The confusion matrix of the Xception model in Fig. 11 demonstrated similarly strong results, with most samples across all four classes correctly predicted: 264 as glioma, 289 as meningioma, 403 as non-tumorous, and 297 as pituitary. The largest source of misclassification was the 33 glioma samples predicted as meningioma. Within the meningioma class, 5 samples were predicted as glioma, bringing the glioma-meningioma confusion errors to a total of 38, 6 as non-tumorous, and 6 as pituitary. These numbers suggest that while Xception performs well for “pituitary” and “no tumor” cases, it is more susceptible to errors between glioma and meningioma, likely due to overlapping features between these tumor types.

The VGG19 confusion matrix in Fig. 12 demonstrates even stronger prediction results than the CNN and Xception models. A total of 296 samples were correctly classified as glioma and another 296 as meningioma. The non-tumorous class showed nearly perfect performance, with 404 samples correctly predicted and only 1 misclassification. The pituitary class had 295 samples correctly predicted. Misclassifications between glioma and meningioma were 10, with only 4 glioma samples classified as meningioma and 6 meningioma samples classified as glioma. These numbers reinforce that VGG19 has the strongest overall predictive performance, with high precision and recall across all classes, and demonstrates a dominant diagonal structure with sparse off-diagonal values in the confusion matrix.

Although the custom-built CNN showed a good level of classification accuracy, it struggled to differentiate between glioma and meningioma tumors, with a significant portion of meningioma cases incorrectly classified as gliomas. Conversely, the Xception model performed well for “pituitary” and “no tumor” cases but showed higher misclassification rates between glioma and meningioma. This affected its class-wise precision for glioma, as a sizable portion of glioma samples were predicted to be meningioma. In contrast, the VGG19 model outperformed the other two, demonstrating consistently accurate classification across all tumor categories. Its sparse off-diagonal values indicate few misclassifications and strong precision and recall for each class.

**Fig. 13:**
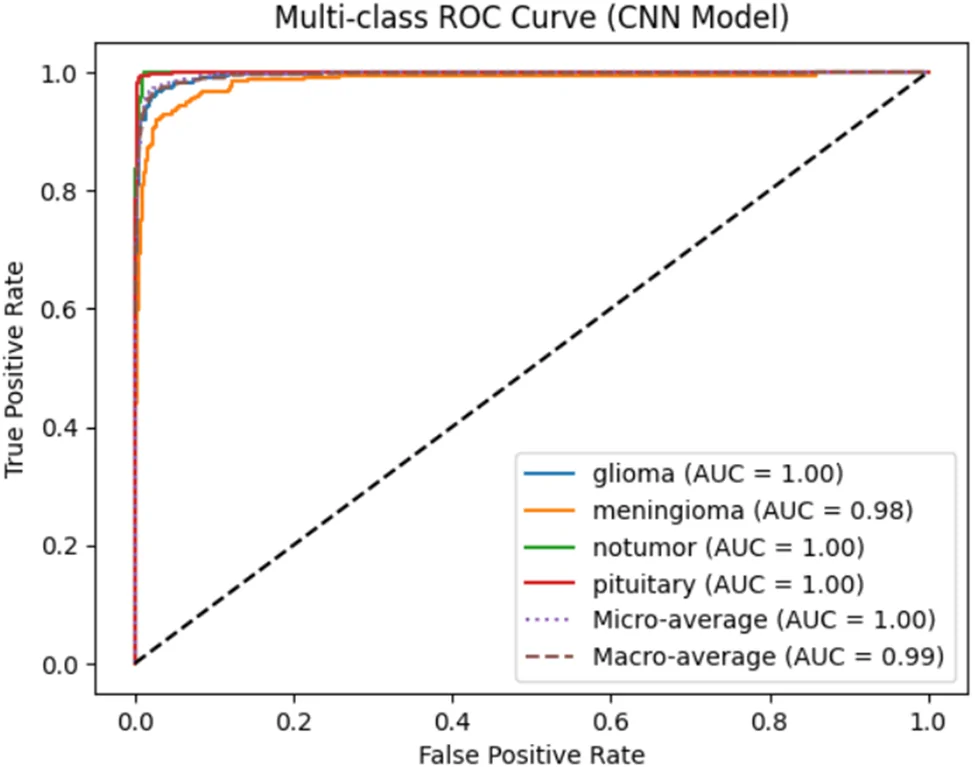
Multi-class ROC curve with AUC values of CNN model

**Fig. 14:**
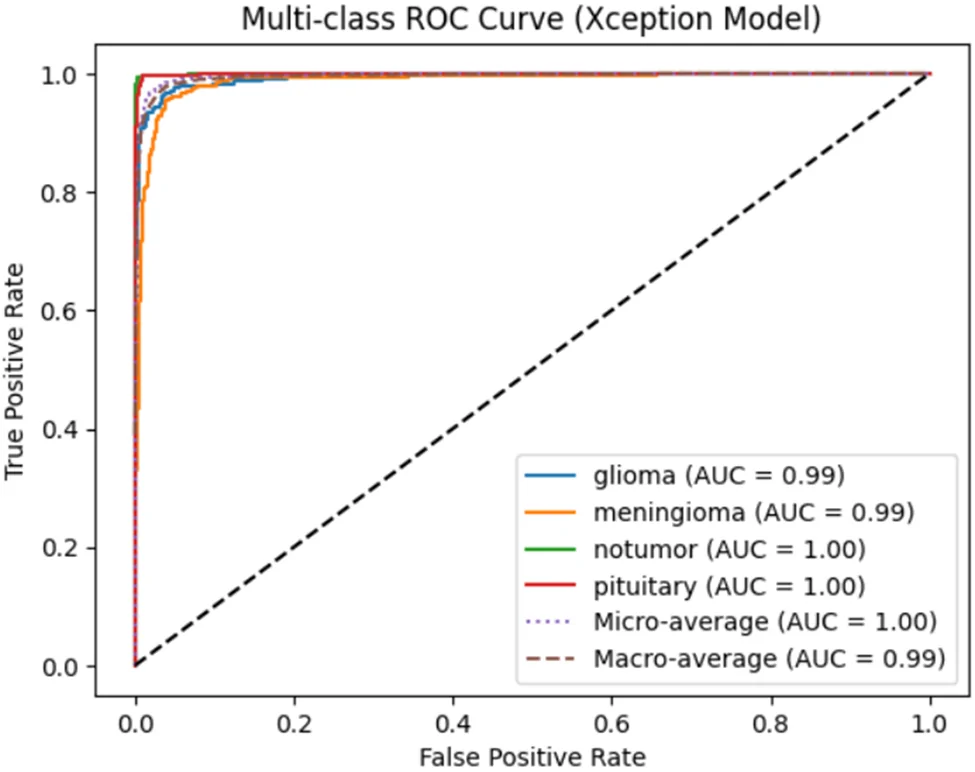
Multi-class ROC curve with AUC values of Xception model

**Fig. 15:**
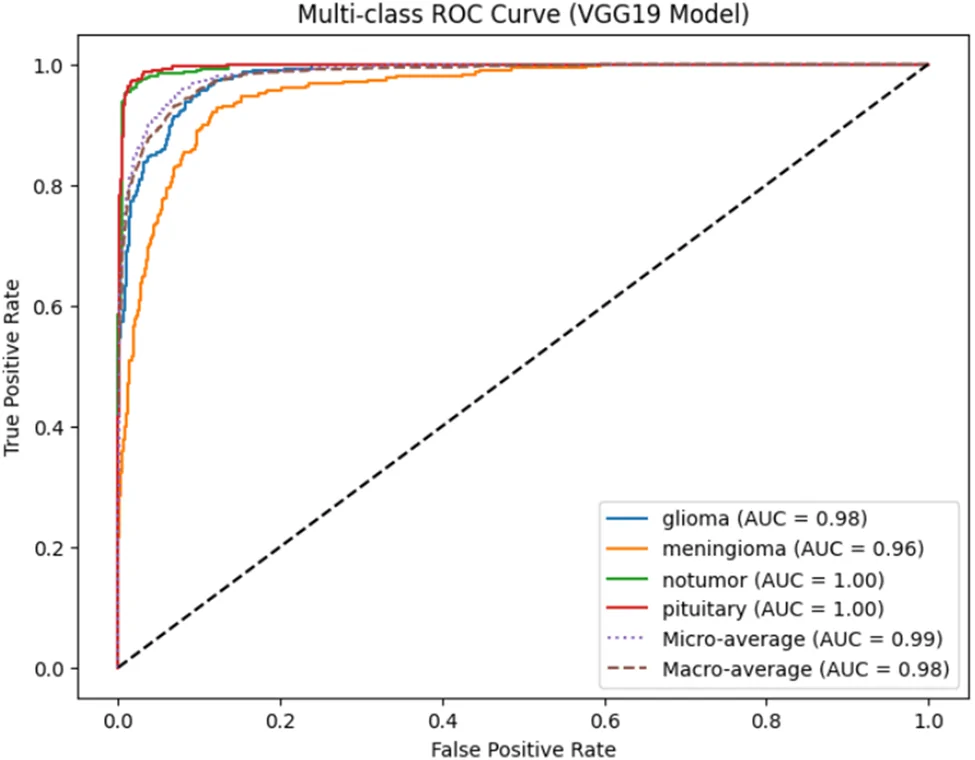
Multi-class ROC curve with AUC values of VGG19 model

In addition, Receiver Operating Characteristic (ROC) curves were generated for each model for multi-class classification purposes. As shown in Fig. 13, the CNN model achieved excellent classification performance across all tumor categories, with AUC values of 0.9954 for glioma, 0.9993 pituitary, 0.9991 no tumor classes, and 0.9836 for meningioma. The micro-average AUC of 0.9955 and macro-average AUC of 0.9945 further confirm the model’s strong overall ability to distinguish between classes. These results indicate that the proposed CNN model is highly effective in capturing class-specific features and achieving robust multi-class separation. However, the slightly lower AUC for the meningioma class suggests comparatively higher classification difficulty, which may be attributed to feature similarity with other tumor types. Overall, the ROC analysis complements the accuracy metrics by demonstrating consistent and reliable performance across different decision thresholds.

As for the ROC results of the Xception model shown in Fig. 14 we can see that it demonstrated excellent discriminative performance across all classes, with AUC values of 0.993 for glioma, 0.9872 for meningioma, 0.9992 for no tumor, and 0.999 for pituitary tumors. The micro-average and macro-average AUC values of 0.9958 and 0.9948, respectively, further confirm the model’s strong overall classification capability.

Finally, Fig. 15 shows the ROC results of the VGG model with AUC values of 0.9814 for glioma, 0.9558 for meningioma, 0.9959 for no tumor, and 0.9971 for pituitary tumors. The micro-average and macro-average AUC values of 0.9861 and 0.9828.s.

While the results are highly promising for each class across all three models, further validation on independent and more diverse datasets is necessary to confirm generalizability in real clinical settings.

Overall, VGG19 was the most reliable and accurate model for classifying brain tumors in this study. Its exceptional performance across all tumor types and low misclassification rates make it the most promising model. However, generalization to external datasets remains invalidated, and performance likely represents an upper bound; clinical deployment would need to address domain shift challenges. For the web-based interface, these results support selecting VGG19 as the default classifier while still offering CNN and Xception as complementary options when faster training or alternative architectural behavior is desired.

#### Error analysis of misclassifications

A closer inspection of the incorrect predictions provides additional insight into the behavior and limitations of the three models. Across the CNN and Xception architectures, most errors arose when attempting to distinguish glioma from meningioma cases. This confusion is not unexpected: when relying solely on single T1-weighted contrast-enhanced slices, the visual characteristics of these two tumors often overlap. Both can appear as enhancing masses with irregular margins, and without complementary MRI sequences, their structural profiles may not be sufficiently distinct for robust classification.

The VGG19 model showed the smallest number of cross-class mistakes, reflecting its stronger feature extraction capability. Nevertheless, the few misclassifications it produced also tended to occur between glioma and meningioma, indicating that this pair represents an intrinsically challenging distinction within the limits of the dataset.

Conversely, mislabeling between tumor categories and the “no tumor” class was uncommon for all three models, suggesting that the classifiers are highly reliable in detecting the presence versus absence of pathology. False negatives were therefore relatively rare in the current setup.

To further quantify model performance, Tables 4 and 5, and 6 show the class-wise metrics that were calculated for each model, including Positive Predictive Value (PPV), Negative Predictive Value (NPV), and False Negative Rate (FNR).

**Table 4:** CNN class metrics

Class	PPV	NPV	FNR
Glioma	0.929	0.989	0.0367
Meningioma	0.958	0.967	0.111
No tumor	0.978	0.993	0.0148
Pituitary	0.971	0.999	0.0033
Average	**0.959**	**0.987**	**0.041**

**Table 5:** VGG19 class metrics

Class	PPV	NPV	FNR
Glioma	0.977	0.996	0.013
Meningioma	0.970	0.990	0.033
No tumor	0.998	0.999	0.0025
Pituitary	0.990	0.995	0.017
Average	**0.984**	**0.995**	**0.010**

**Table 6:** Xception class metrics

Class	PPV	NPV	FNR
Glioma	0.978	0.965	0.120
Meningioma	0.887	0.983	0.056
No tumor	0.985	0.998	0.0049
Pituitary	0.971	0.997	0.010
Average	**0.955**	**0.986**	**0.048**

Overall, these findings highlight a well-known clinical reality: accurately separating gliomas from meningiomas typically requires information from multiple MRI sequences, such as FLAIR or T2, which better capture edema extent, infiltration patterns, and peritumoral changes. The reliance on a single contrast-enhanced sequence restricts the models’ ability to leverage these discriminative cues. Future versions of the platform will incorporate multi-sequence data fusion to mitigate this limitation and improve diagnostic specificity.

**Fig. 16:**
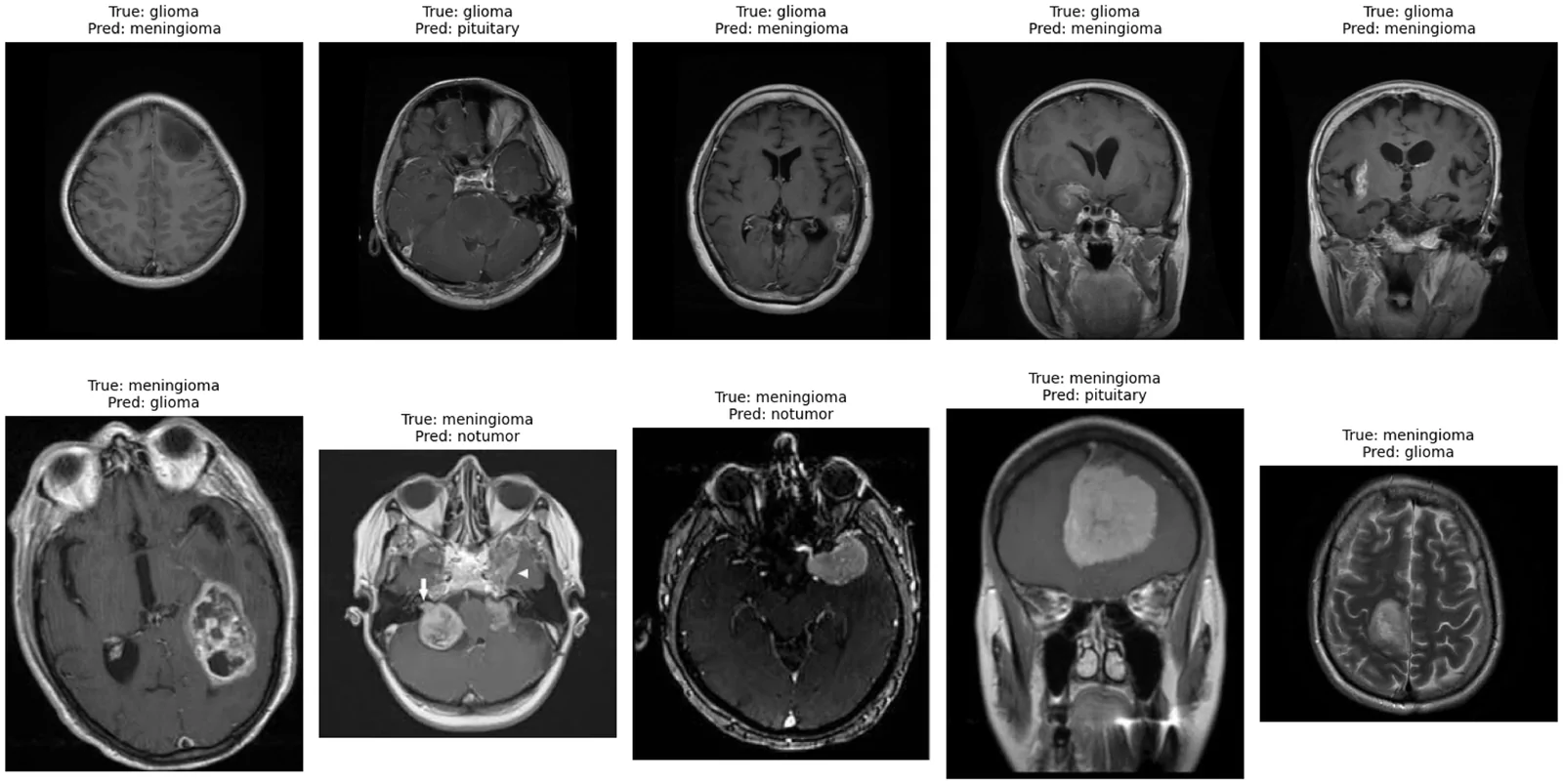
CNN model misclassifications of glioma and meningioma

**Fig. 17:**
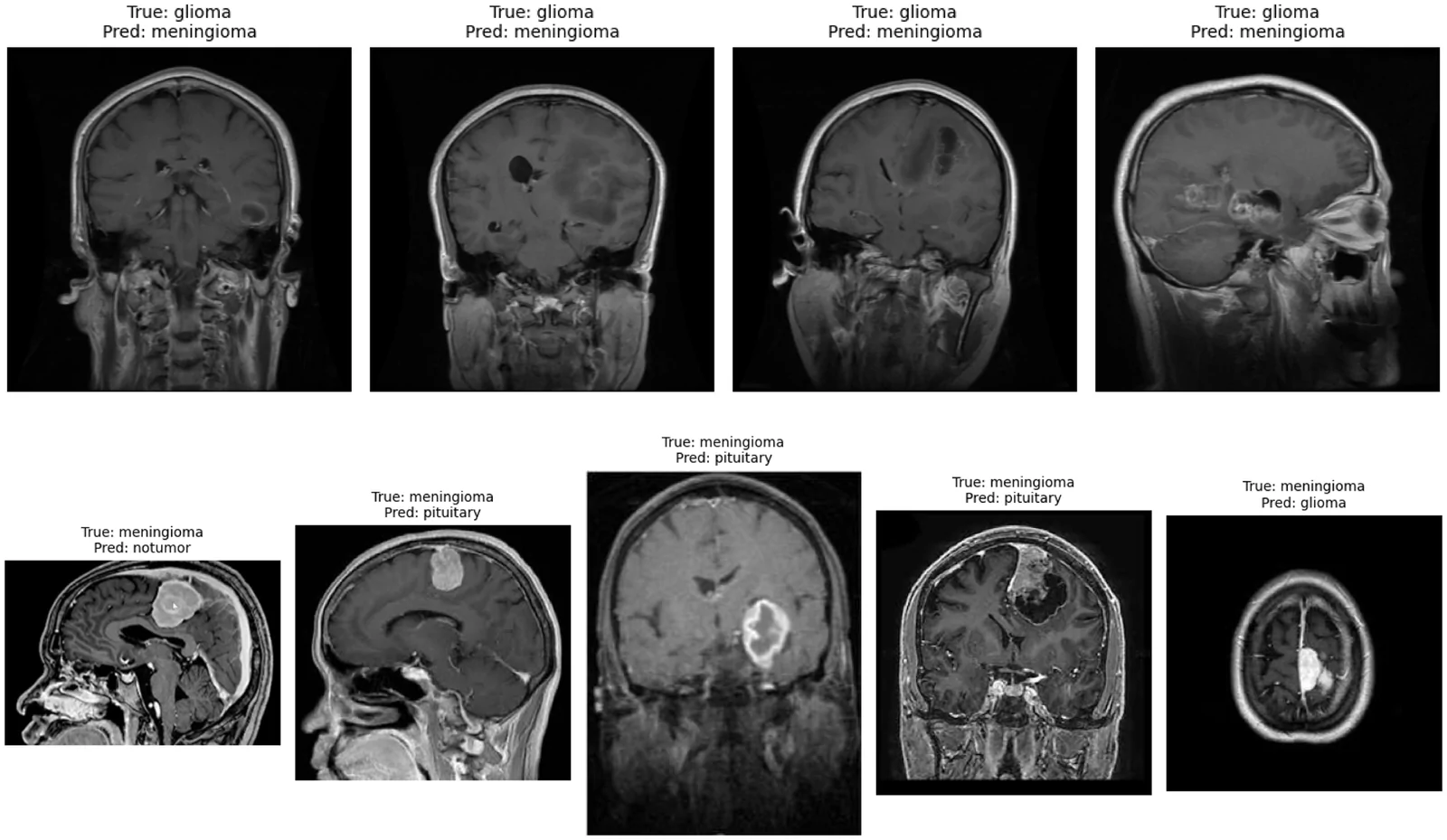
VGG19 model misclassifications of glioma and meningioma

**Fig. 18:**
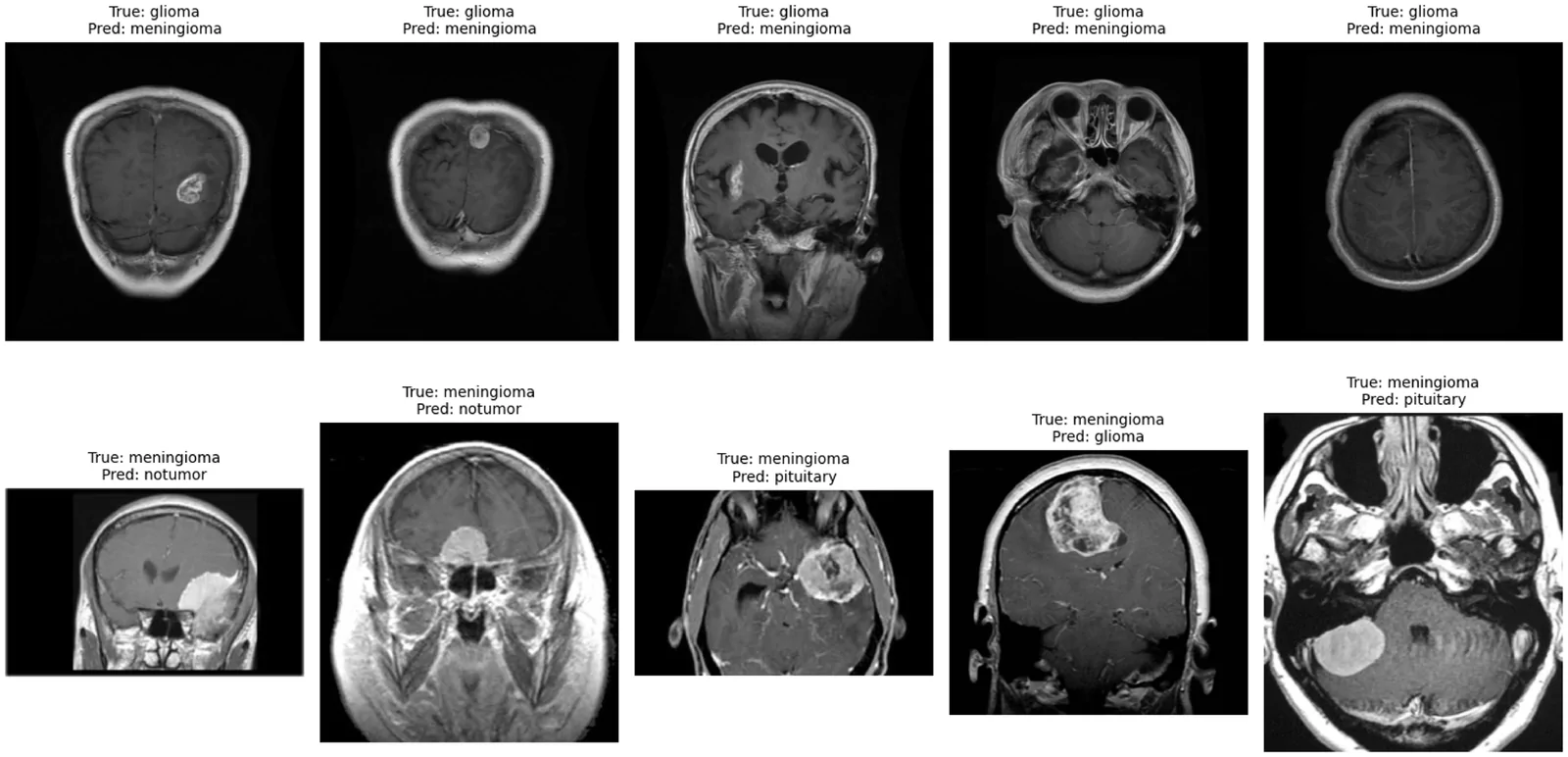
Xception model misclassifications of glioma and meningioma

For qualitative purposes, representative MRI slices with their true and predicted labels are provided in Figs. 16, 17 and 18 for each model to shed light on classification behavior and show which specific cases were misclassified.

Across all three non-augmented models (Custom CNN, VGG19, and Xception), misclassifications were predominantly observed between glioma and meningioma cases. Visual inspection of the misclassified images suggests that tumors with smooth, well-defined margins and relatively homogeneous enhancement were more likely to be confused, particularly when gliomas presented with round or peripheral appearances that resemble typical meningioma characteristics. Several errors also involved lesions located near the skull boundary, where extra-axial and intra-axial distinctions may be less visually pronounced in slice-based analysis. In addition, smaller or less conspicuous tumors appeared more prone to incorrect classification, likely due to reduced discriminative texture and contextual features. Overall, the observed errors indicate that the models rely strongly on shape and intensity-based features, and that overlapping radiological characteristics between glioma and meningioma remain the primary source of confusion.

#### Training dynamics

**Fig. 19:**
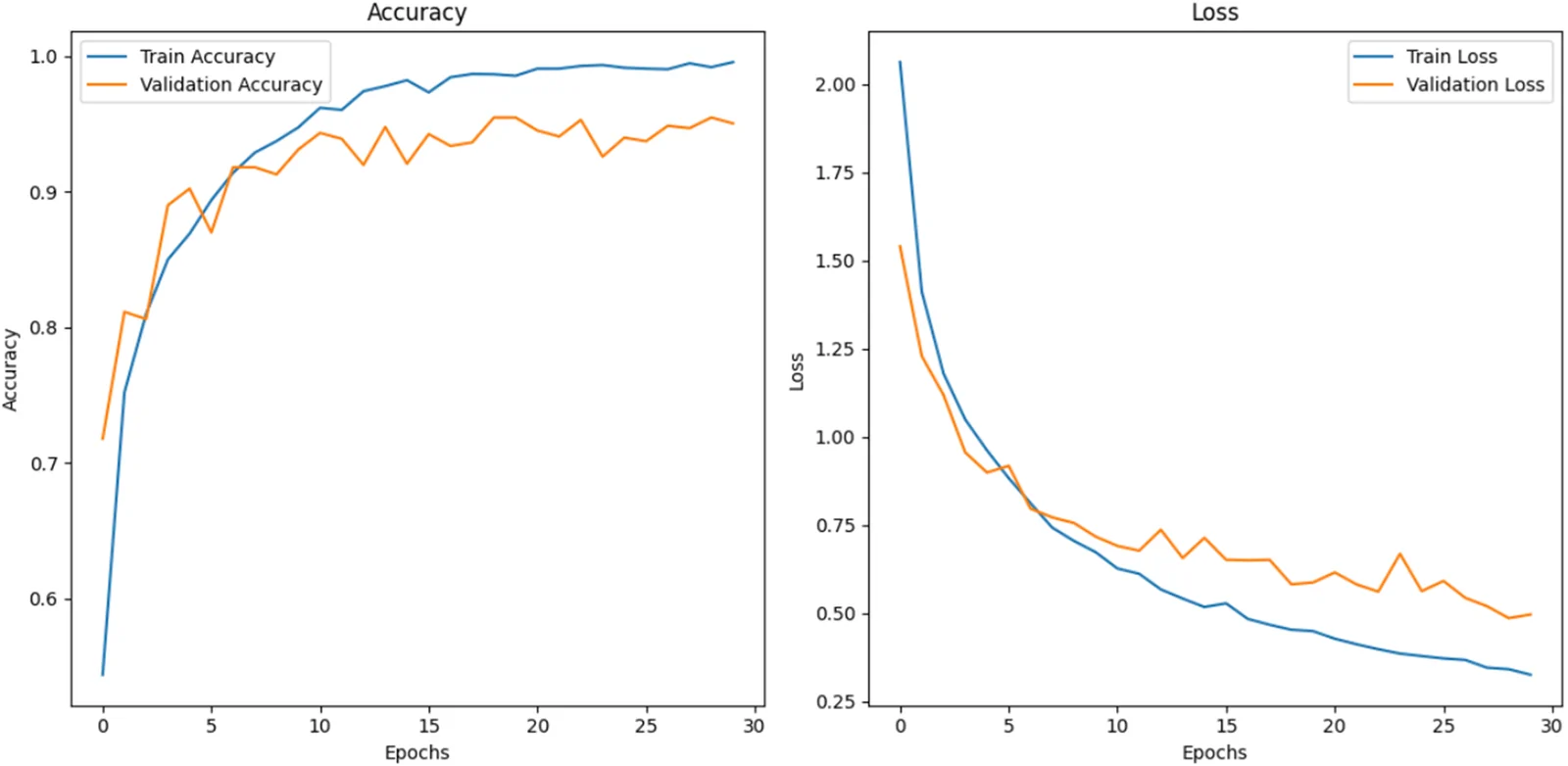
CNN model learning curves

**Fig. 20:**
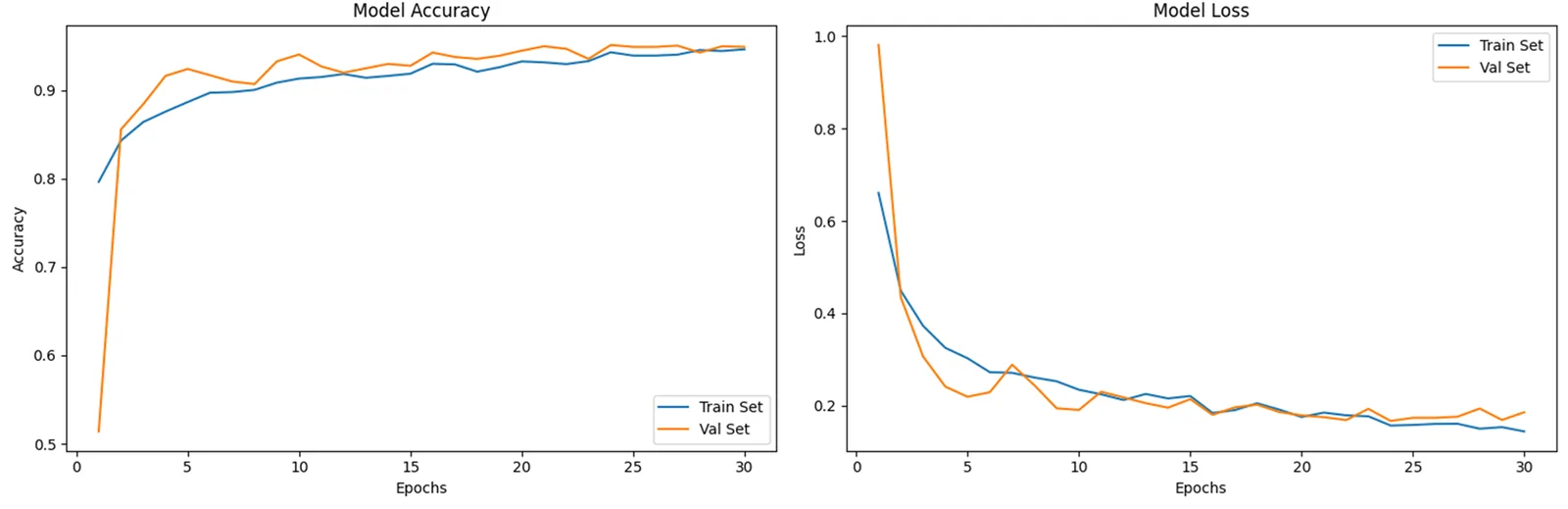
VGG19 model learning curves

**Fig. 21:**
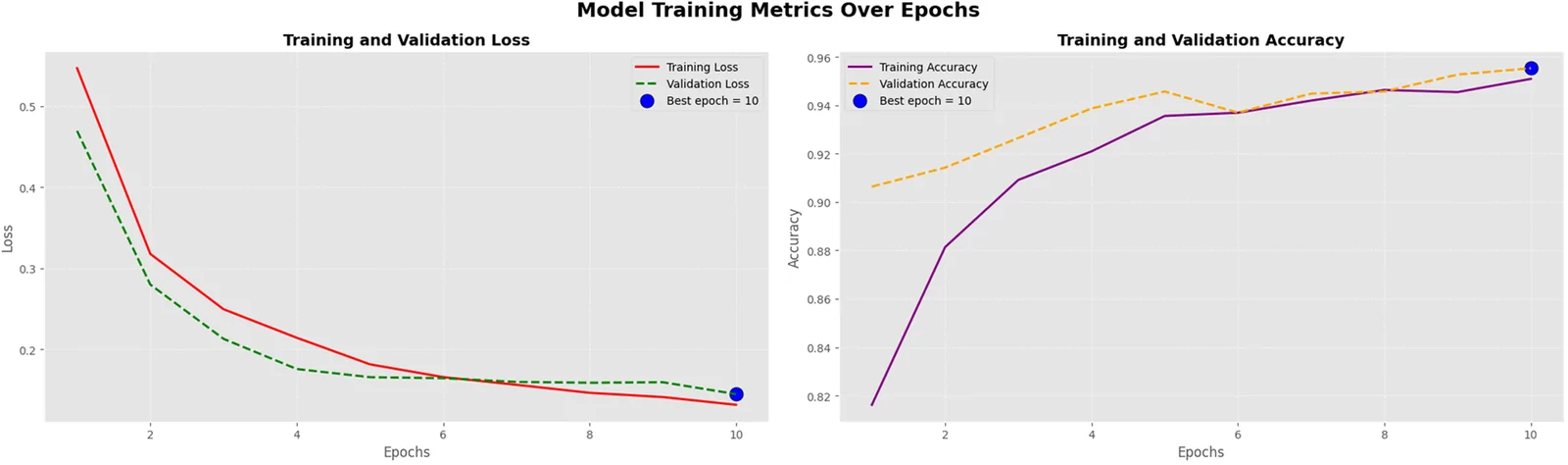
Xception model learning curves

Learning curves (accuracy and loss vs. epoch) shown in Figs. 19, 20 and 21 revealed distinct convergence behaviors.

- CNN: Converged rapidly within 10 epochs, with minor oscillations between training and validation accuracy, suggesting slight overfitting.
- VGG19: Achieved stable convergence around 20 epochs, with parallel training/validation trends and minimal divergence, reflecting robust learning.
- Xception: Reached plateau within 8–10 epochs, maintaining moderate variance due to smaller effective training depth after layer freezing.

Overall, the pretrained architectures exhibited smoother loss decay and better validation consistency, confirming the benefit of transfer learning for medical image tasks with limited datasets.

#### Comparative analysis of preprocessing variants

A study was conducted across three architectures: a custom CNN, VGG19, and Xception. Each architecture was evaluated with and without preprocessing under identical training conditions. Table 7 showcases the results obtained for each model in each case.

**Table 7:** Comparative results with vs. without preprocessing

Model	Preprocessing	Test Accuracy (%)	Precision (%)	Recall (%)	F1-Score (%)
CNN	Yes	96.00	96.00	96.00	96.00
CNN	No	95.7	96.00	95.00	95.00
VGG19	Yes	98.00	98.00	98.00	98.00
VGG19	No	97.00	96.00	96.00	96.00
Xception	Yes	96.00	96.00	95.00	95.00
Xception	No	88.59	89.76	87.50	88.61

The CNN model achieved 96.0% accuracy with preprocessing and 95.7% without it, indicating only a negligible difference. This suggests that the CNN is relatively robust to input distribution changes, likely because it learns task-specific features from scratch.

Similarly, VGG19 demonstrated stable performance, with 98.0% accuracy when preprocessing was applied and 97.0% without it. Although a slight decrease is observed, the model remains largely unaffected, reflecting the resilience of deep pretrained convolutional features even under mild distribution shifts.

In contrast, Xception exhibited a substantial performance drop from 96.0% with preprocessing to 88.59% without preprocessing. This highlights a strong dependency on proper input normalization consistent with its ImageNet pretraining. The depthwise separable convolution architecture appears more sensitive to deviations in input distribution, making preprocessing a critical factor for maintaining performance.

Overall, the findings indicate that preprocessing effects are architecture-dependent. While simpler or moderately deep models such as CNN and VGG19 show robustness to input variations, more advanced transfer learning architectures like Xception are highly sensitive to preprocessing consistency. This suggests that model complexity and reliance on pretrained feature distributions increase the importance of proper input normalization.

In conclusion, the impact of preprocessing varies across model architectures. While the custom CNN demonstrated relatively stable performance with and without preprocessing, deeper transfer learning models, particularly Xception, showed greater sensitivity to preprocessing strategies. Recent studies have similarly reported that preprocessing significantly influences model convergence, feature extraction quality, and overall classification performance in medical image analysis [41–43]. Our findings suggest that preprocessing becomes increasingly important as architectural complexity and dependency on pretrained feature representations increase, highlighting the need to tailor preprocessing pipelines to the selected model architecture.

**Fig. 22:**
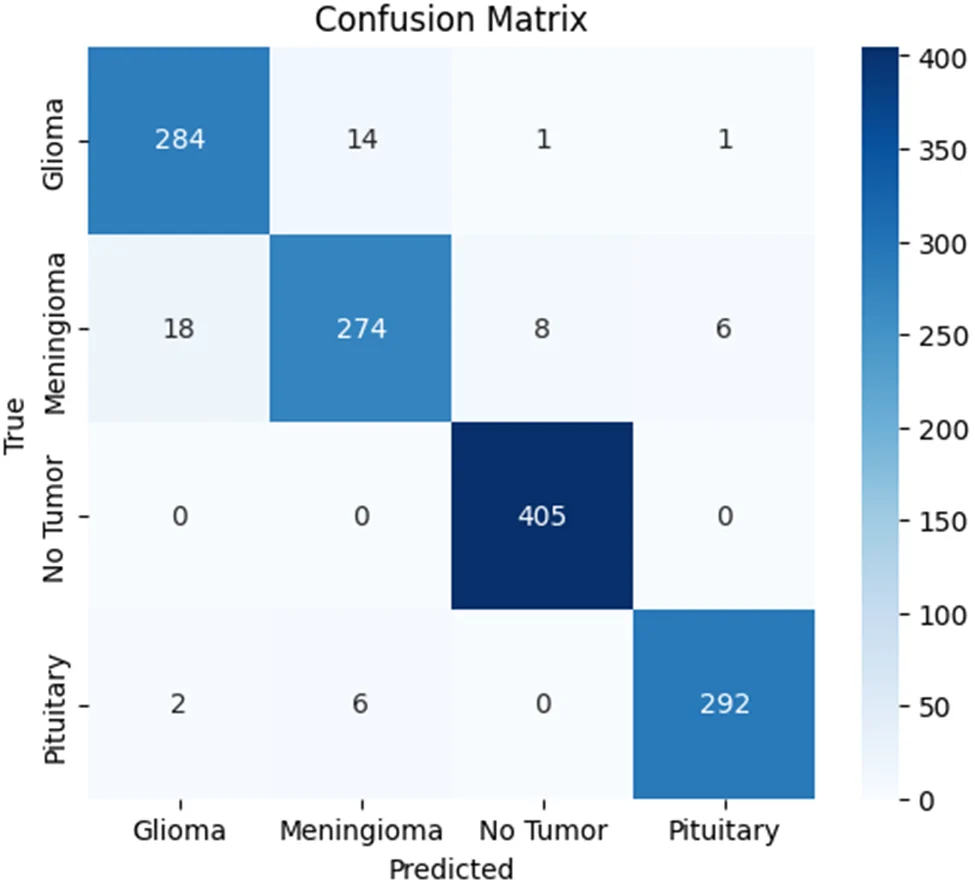
CNN results with no preprocessing as a confusion matrix

**Fig. 23:**
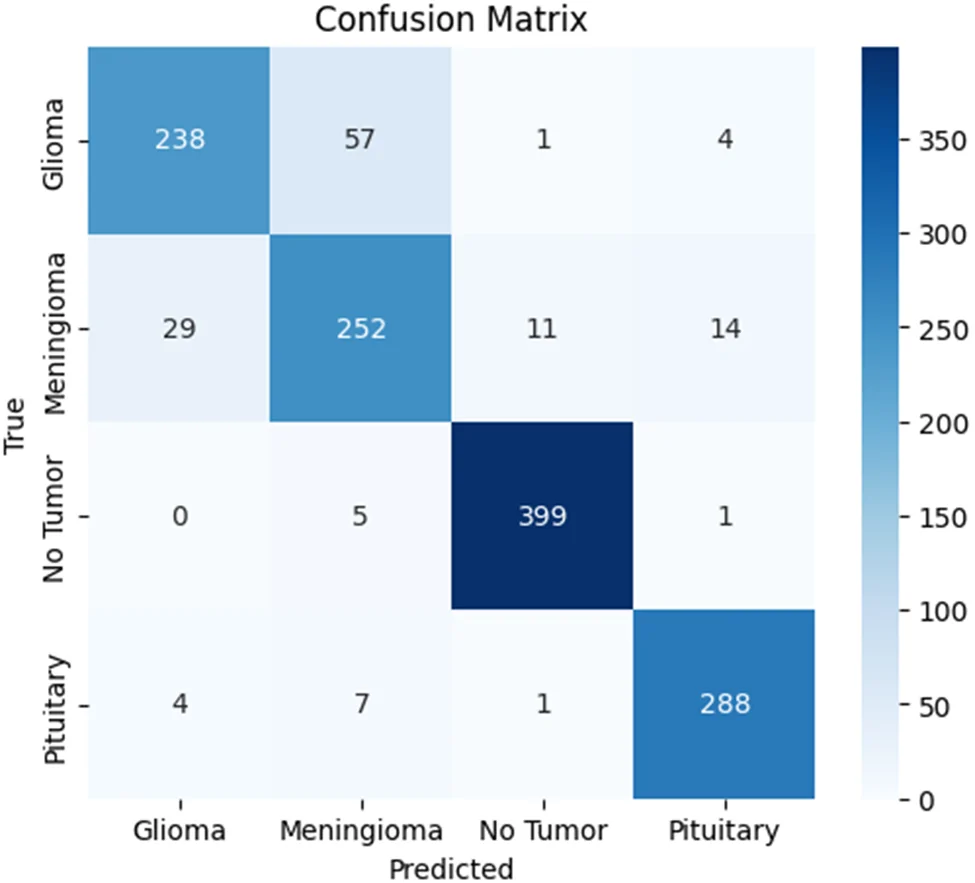
Xception results with no preprocessing as a confusion matrix

**Fig. 24:**
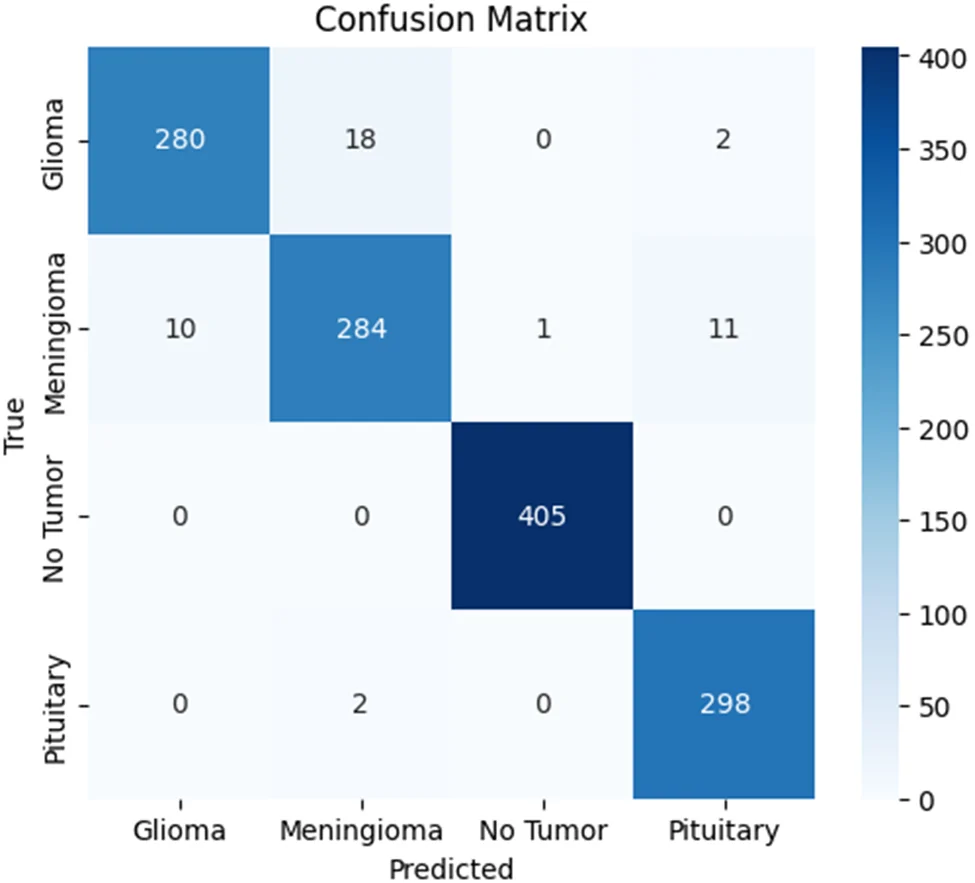
VGG19 results with no preprocessing as a confusion matrix

Confusion matrices were included in Figs. 22 and 23, and 24 to further analyze class-wise prediction behavior under different preprocessing conditions. They confirm that performance variations, particularly in Xception, are associated with increased inter-class misclassification rather than uniform accuracy degradation.

### Models’ performance overview with augmentation

To assess the impact of data augmentation on model performance, the same experimental setup was repeated using online augmentation during training. Augmentation strategies included geometric and intensity-based transformations such as rotation, flipping, zooming, and minor intensity variations, with the goal of improving model robustness and reducing overfitting.

**Table 8:** Quantitative models’ performance overview with augmentation

Evaluation Metrics	Custom CNN	VGG19	Xception
Train Accuracy (%)	90.69	85.10	95.22
Validation Accuracy (%)	91.87	91.08	95.36
Test Accuracy (%)	91.15	86.80	94.28
Precision (%)	91.11	86.76	93.74
Recall (%)	91.15	86.79	93.09
F1-Score (%)	91.03	86.57	93.41
Epochs	30	30	10
Time (min)	135	69	197
Mean Inference Time (s/image)	0.0076	0.01435	0.1927

As shown in Table 8, the augmented training experiments reveal that the impact of data augmentation varied across model architectures. Quantitatively, the augmented CNN and Xception models maintained high test accuracies of 91.15% and 94.28%, respectively, indicating reasonable results under augmented conditions. However, the augmented VGG19 model exhibited a substantial degradation in performance, with test accuracy dropping to 86.80%, suggesting that the applied augmentation strategy may have introduced excessive variability and reduced the model’s ability to generalize effectively under the current training configuration.

**Fig. 25:**
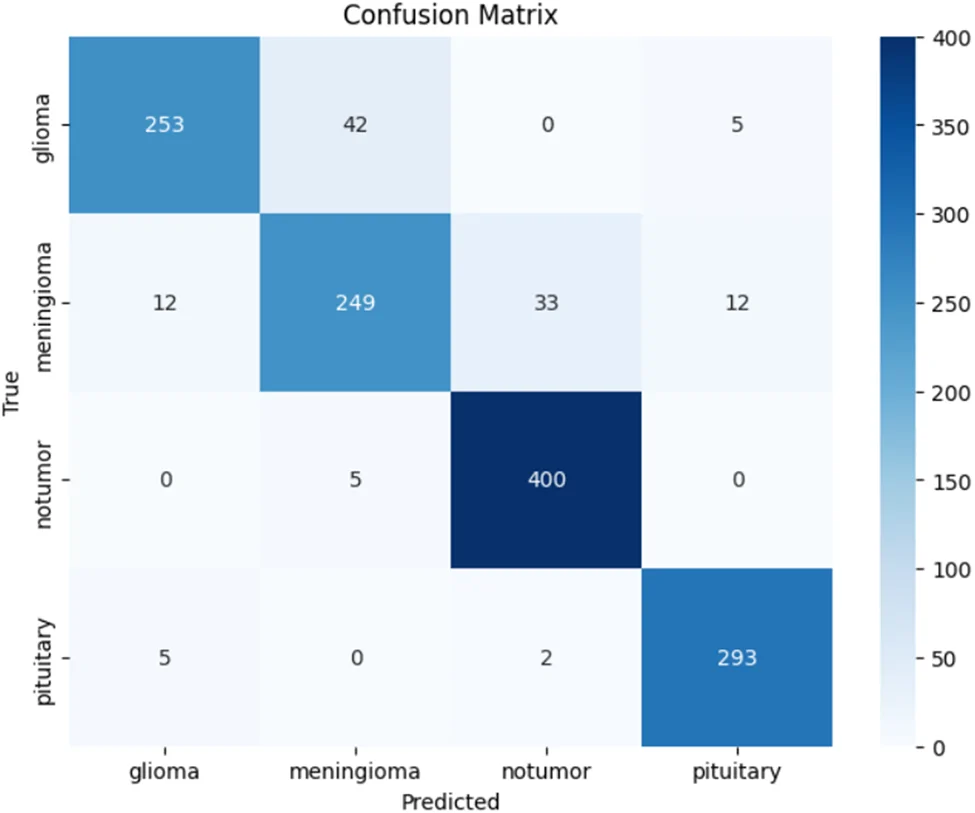
Augmented CNN results as a confusion matrix

**Fig. 26:**
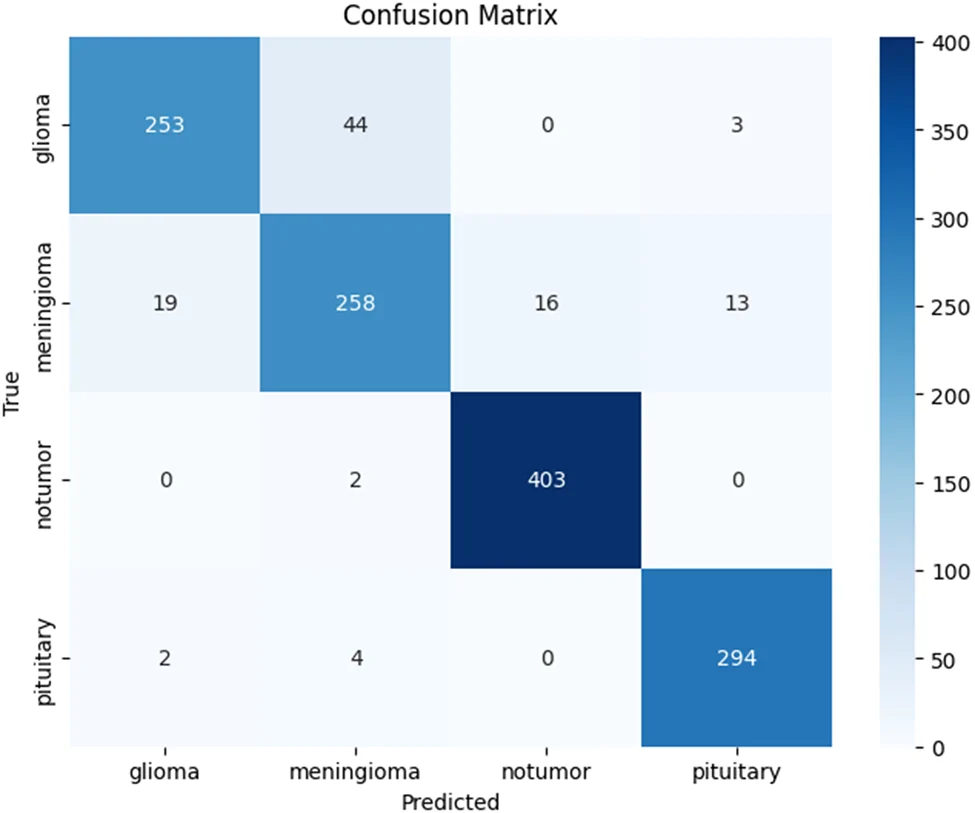
Augmented Xception results as a confusion matrix

**Fig. 27:**
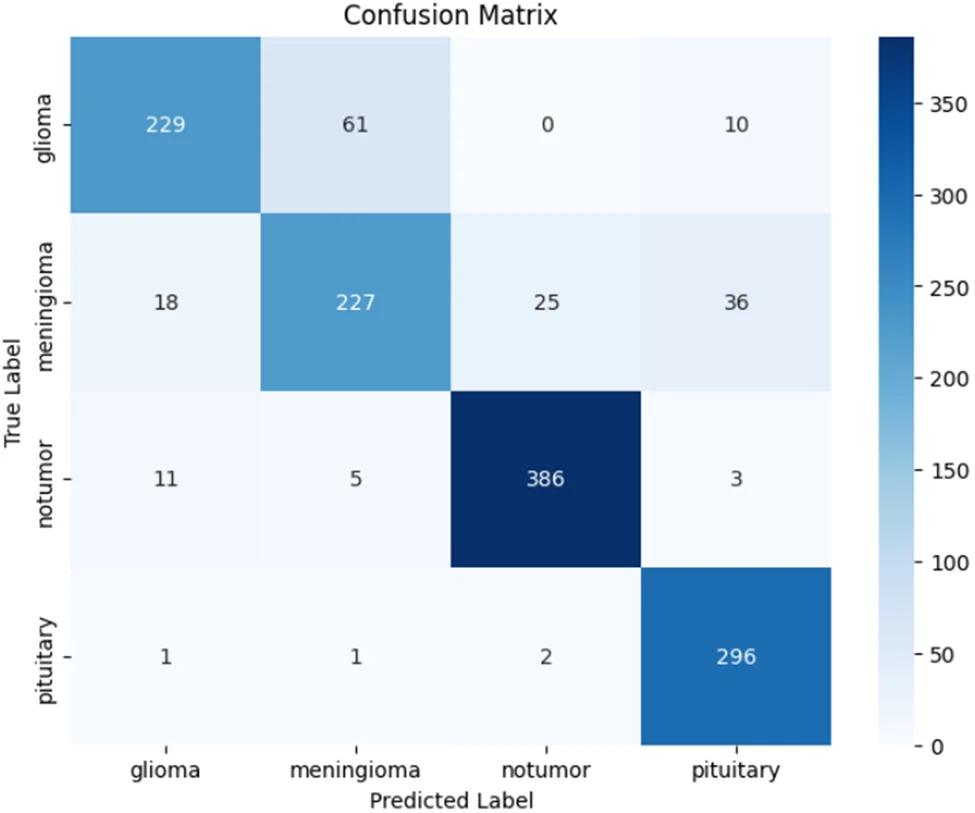
Augmented VGG19 results as a confusion matrix

Analysis of the confusion matrices shown in Figs. 25 and 26, and 27 further indicates increased off-diagonal errors, especially for glioma and meningioma classes, pointing to higher misclassification rates under augmentation. For the CNN and Xception models, augmentation improved training stability but did not consistently translate into improved test performance. In contrast, the VGG19 architecture appeared particularly sensitive to the augmented data. Aside from the increase in meningioma-glioma errors, there appeared to be some misclassifications of the no tumor class as well. We hypothesize that the observed performance collapse may be due to a combination of factors, including the use of a frozen VGG19 feature extractor and a potential distribution shift introduced by overly aggressive combined augmentations. However, this remains to be experimentally validated and will be further investigated through controlled fine-tuning and augmentation studies.

Overall, the augmented results demonstrate that while data augmentation can enhance robustness in certain architectures, it does not universally improve performance and may, in some cases, negatively affect classification reliability.

### Ablation study

To analyze the contribution of different data augmentation strategies on performance, an ablation study was conducted on each of the three models separately. The objective of this analysis is to isolate the effect of each augmentation technique and quantify its impact on brain tumor MRI classification. Each experiment was trained under identical conditions, with only the augmentation strategy varying, ensuring a fair comparison across configurations. The evaluated augmentation techniques include rotation, translation (shift), zoom, brightness adjustment, and horizontal flipping. In addition, there will be a comparison between the results where all transformations were applied. The model was trained separately for each configuration, and performance was assessed based on accuracies, precision, recall, and F1-score. The results are shown in Tables 9 and 10, and 11.

#### CNN ablation study

**Table 9:** CNN ablation study results

Augmentation Type	Test Accuracy (%)	Precision (%)	Recall (%)	F1-Score (%)
Rotation	92.91	93.07	92.89	92.65
Shift	84.97	85.47	84.95	84.01
Zoom	92.74	92.70	92.75	92.63
Brightness	92.30	92.38	92.32	92.23
Flip	94.74	94.79	94.72	94.68
All Combined	91.15	91.11	91.17	91.03

Among the evaluated methods, horizontal flipping achieved the highest performance, obtaining a test accuracy of 94.74%, precision of 94.79%, recall of 94.72%, and F1-score of 94.68%. Rotation and zoom augmentations also produced strong results, with accuracies of 92.91% and 92.74%, respectively, indicating that moderate spatial transformations improved the model’s robustness to variations in tumor orientation and scale. Brightness augmentation similarly enhanced performance, suggesting that intensity-based variations contributed positively to generalization across MRI scans acquired under different imaging conditions. In contrast, shift augmentation yielded the lowest performance, with an accuracy of 84.97%, likely because excessive spatial translation may disrupt the anatomical alignment and structural consistency of brain MRI images. Interestingly, the combined augmentation pipeline achieved lower performance than several individual augmentations, suggesting that excessive or compounded transformations may introduce unnecessary variability and distort clinically relevant image features. Overall, the findings indicate that carefully selected augmentation strategies can improve CNN-based brain tumor classification more effectively than applying multiple augmentations simultaneously.

#### Xception ablation study

**Table 10:** Xception ablation study results

Augmentation Type	Test Accuracy (%)	Precision (%)	Recall (%)	F1-Score (%)
Rotation	93.44	93.49	93.43	93.37
Shift	92.75	92.71	92.76	92.65
Zoom	93.36	93.42	93.37	93.33
Brightness	96.19	96.20	96.19	96.18
Flip	95.35	95.41	95.33	95.34
All Combined	94.28	93.74	93.09	93.41

The ablation study results showed that different augmentation strategies influenced Xception performance to varying degrees, although the model remained consistently robust across all experiments. Brightness augmentation achieved the highest performance, reaching 96.19% accuracy, indicating that intensity-based variations improved the model’s ability to generalize across MRI scans with different contrast characteristics. Horizontal flipping also produced strong results (95.35%), while rotation and zoom augmentations achieved similarly high performance above 93%, demonstrating the effectiveness of moderate geometric transformations in improving feature invariance. Unlike the CNN model, shift augmentation did not significantly reduce performance, suggesting that Xception possesses stronger spatial feature extraction capabilities and greater robustness to positional variations. Although the combined augmentation pipeline achieved strong overall performance, several individual augmentations outperformed it, indicating that carefully selected augmentation strategies may be more beneficial than applying multiple augmentations simultaneously.

#### VGG19 ablation study

**Table 11:** VGG19 ablation study results

Augmentation Type	Test Accuracy (%)	Precision (%)	Recall (%)	F1-Score (%)
Rotation	89.17	89.21	89.15	88.96
Shift	88.48	88.53	88.46	88.29
Zoom	87.87	87.91	87.85	87.58
Brightness	91.84	91.80	91.85	91.74
Flip	91.08	91.01	91.10	90.98
All Combined	86.80	86.76	86.79	86.57

The ablation study conducted on the VGG19 demonstrates that different augmentation strategies have a noticeable but consistent impact on classification performance. Among the evaluated techniques, brightness achieved the highest performance across all metrics, with a test accuracy of 91.84% and the strongest precision, recall, and F1-score values, indicating that intensity variations significantly improve the model’s ability to generalize to MRI scans with varying contrast conditions. Horizontal flipping also yielded strong results (91.08%), suggesting that spatial orientation is not highly discriminative for the dataset and that symmetry-based augmentation enhances robustness. In contrast, rotation, shift, and zoom augmentations produced slightly lower but stable performance, ranging between 87% and 89%, indicating that excessive geometric transformations may moderately affect the preservation of structural tumor features. Notably, the combined augmentation strategy resulted in the lowest performance (86.80%), suggesting that applying multiple transformations simultaneously may introduce excessive variability and a distribution shift that negatively affects the model’s ability to learn meaningful representations. Overall, these findings indicate that VGG19 benefits more from targeted augmentation techniques, particularly brightness and flipping.

In conclusion, the ablation study across CNN, VGG19, and Xception shows that data augmentation improves performance, but its effectiveness depends on both the model and the type of augmentation [44]. Xception is the most robust and consistently performs best [45], VGG19 benefits mainly from brightness and flipping, while the CNN is the most sensitive to augmentation changes. Overall, the best results are obtained using carefully selected, individual augmentation strategies rather than combining all transformations. These findings are consistent with recent literature reporting that data augmentation in medical imaging contributes differently to model performance depending on the type and intensity of transformations [46], and that overly aggressive augmentation may distort clinically relevant image features and introduce distribution shifts that negatively affect generalization [47]. Furthermore, existing studies emphasize that the effectiveness of augmentation strategies is not uniform across architectures or tasks [48], highlighting that augmentation is not universally transferable and requires careful design and systematic ablation-based evaluation.

### Comparative analysis: with vs. without augmentation

A clear performance difference is observed between the augmented and non-augmented training setups. Without data augmentation, all three models achieved strong and stable results, with test accuracies of 96.03% for the CNN, 98.00% for VGG19, and 96.00% for Xception. In contrast, applying data augmentation led to improved robustness for the CNN and Xception models, which maintained high test accuracies of 91.15% and 94.28%, respectively, but caused a degradation in VGG19 performance, where test accuracy dropped to 85.10%. This suggests that while augmentation benefited or preserved performance in lighter and more modern architectures, the VGG19 model was unable to adapt as effectively to the augmented data under the current training configuration.

One likely explanation is that the VGG19 backbone was used as a frozen feature extractor, meaning its convolutional layers were not updated during training. When augmentation introduced geometric and intensity variations (e.g., rotations, shifts, or brightness changes), the frozen filters—originally optimized for natural images—were unable to adapt to the new distribution of medical features. This augmentation–feature mismatch can lead to poor feature representation and unstable classification. Additionally, VGG19’s deeper architecture and large parameter count may make it more sensitive to distribution shifts when only the classifier head is trained, causing underfitting or feature misalignment.

Based on these results, the non-augmented models were selected for all subsequent analyses, as they provided more consistent and reliable performance. In future work, this issue will be addressed by progressive fine-tuning of the upper convolutional blocks of VGG19 to allow adaptation to augmented MRI features, along with revision of the augmentation pipeline to ensure clinically realistic transformations (e.g., moderate rotations, controlled intensity scaling, and avoidance of excessive spatial distortion). This approach is expected to improve robustness while preserving diagnostic feature integrity.

### Comparison with previous studies

Now for further analysis, Table 12 presents a comparative benchmark analysis between our three models and a few other models from already published studies that were also trained and tested on the same dataset. Although Disci et al. (2025) used the same publicly available brain tumor MRI dataset, their paper does not specify whether the predefined train–test split provided by the dataset was used. Consequently, differences in dataset partitioning, preprocessing, and augmentation strategies may account for variations in reported performance, and direct numerical comparison should be interpreted with caution.

**Table 12:** Benchmark comparison with existing literature

Models	Test Accuracy (%)	Precision (%)	Recall (%)	F1-Score (%)	Reference
**Our CNN**	96.03	96.00	96.00	96.00	This study
**Our VGG19**	98.02	98.00	98.00	98.00	This study
**Our Xception**	96.00	96.00	95.00	95.00	This study
MobileNetV2	94.5	94.26	94.04	94.11	Disci 2025 [49]
InceptionV3	94.5	94.00	94.00	94.00	Disci 2025 [49]
ResNet50	90.62	91.78	89.93	90.10	Disci 2025 [49]
VGG16	95.04	94.77	94.7	94.7	Disci 2025 [49]
DenseNet121	92.85	92.44	92.30	92.20	Disci 2025 [49]

Based on these percentages, we can see that our models performed better than a number of well-known deep learning models. The model with the best results remained to be our VGG19, with a test accuracy of 98.02%, and the same percentage for precision, recall and f1-score. Our custom CNN model also outperformed the other models with its 96.03% test accuracy and 96% across all (96%), evaluation metrics. Even with the slightly lower recall and f1-score (95%), and test accuracy (96%), our Xception model still performed better than the rest, especially since the other models seemed to have results ranging between 90 and 95% across the accuracy and all the other evaluation metrics listed in the table.

These findings validate the robustness of the models created in this project for brain tumor classification, in turn supporting the efficiency of both transfer learning and custom-built architectures when combined with appropriate training strategies. Moreover, demonstrating performance that is competitive with or superior to prior work strengthens the case for packaging these models into a single modular web platform that can be directly explored and compared by clinicians in routine practice.

### Clinical translation and deployment considerations

Despite promising performance, several challenges must be addressed before clinical deployment. Integration into routine workflows would require compatibility with existing hospital systems, including PACS and RIS, with support for DICOM-formatted images, HL7 messaging, and compliance with data security and privacy regulations.

The platform is intended as a clinical decision support tool to assist radiologists, rather than an autonomous diagnostic system. Potential use cases include second-opinion support, workflow triage, and research or educational applications, with final diagnostic responsibility remaining with the clinician.

Clinical use would also raise considerations related to liability, user training, and regulatory approval. Deployment would require certification as a medical device, such as FDA 510(k) clearance or CE marking.

In summary, real clinical translation will require external validation with patient-level splits across multi-center datasets to ensure generalizability, integration with clinical systems such as DICOM/PACS and HL7 for seamless workflow adoption, and regulatory clearance supported by thorough clinical evaluation and documentation.

## Web-based platform integration

### System architecture

The trained models were deployed within a modular web-based architecture designed for real-time medical image analysis. The platform adopts a client–server structure composed of a React.js frontend and a FastAPI backend implemented in Python. The frontend facilitates user interaction, multilingual support, and visualization of model outputs, while the backend handles API routing, model loading, image preprocessing, and inference execution.

The workflow begins when a user uploads an MRI image through the frontend interface. The backend preprocesses the input image and loads the selected trained model to perform inference. The resulting predictions, including tumor class labels and confidence scores, are returned to the frontend as structured JSON responses. These results are then presented to the user, with the option to generate a downloadable PDF report summarizing the analysis.

**Fig. 28:**
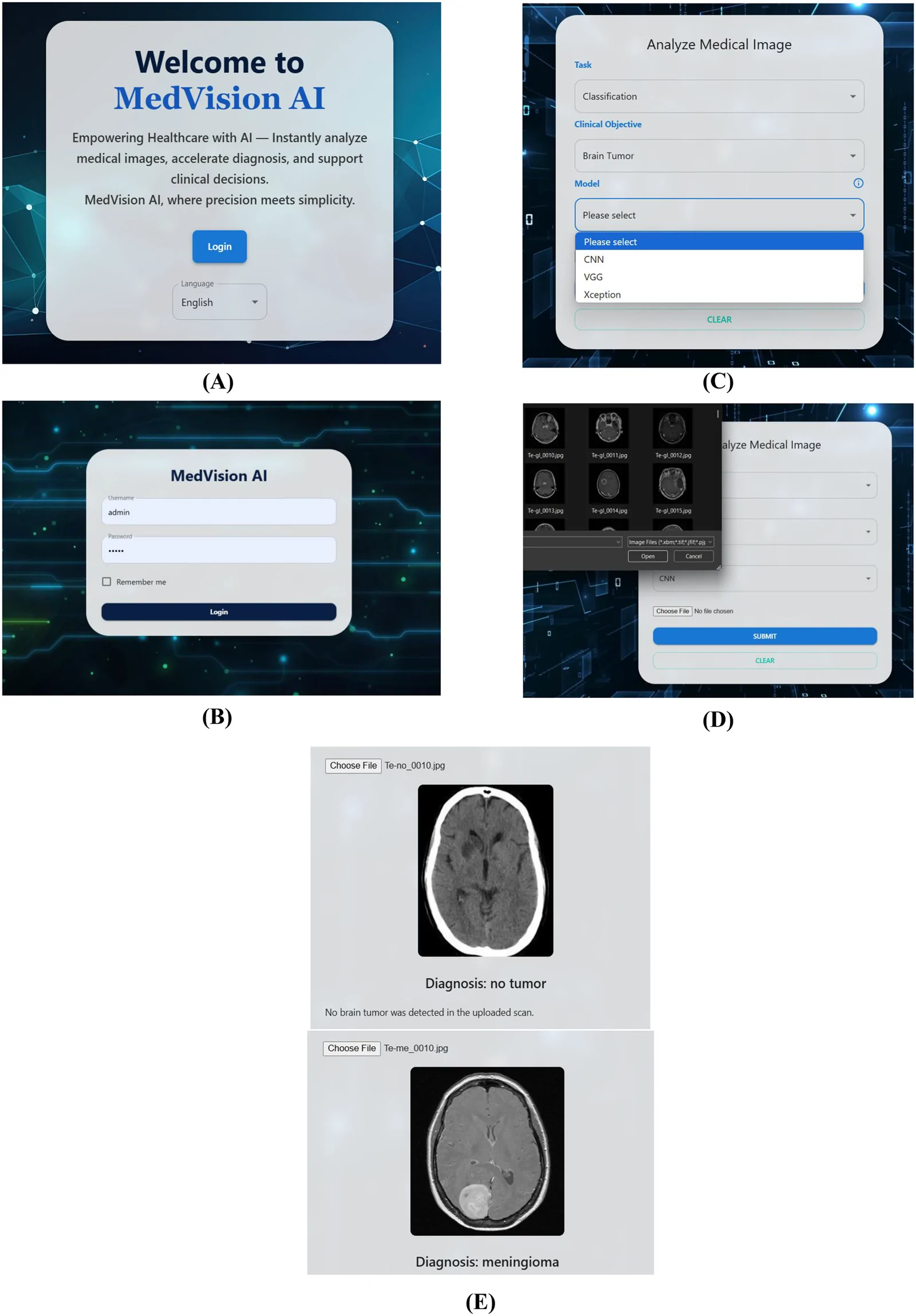
illustrates the graphical user interface of the proposed MedVision AI platform, highlighting the key stages of user interaction from platform entry to prediction output

Figure 28. *Showcase of the web-interface platform showing (A) The welcome page of our platform MedVision AI*,* (B) The login page*,* (C) The analysis page with the needed tasks*,* clinical objective*,* and model selection*,* (D) The image upload panel*,* (E) The prediction output.*

### Modular design

The backend is implemented using a RESTful API built with FastAPI and designed to support modular AI workflows. The API exposes endpoints for querying available tasks, clinical objects, and model options, enabling dynamic configuration from the frontend.

Model versioning is handled implicitly through model identifiers and file-based storage, allowing multiple trained models to coexist and be loaded on demand. Loaded models are cached in memory to reduce inference latency and avoid redundant disk access.

Uploaded image data are processed in-memory and are not permanently stored on the server, limiting exposure of potentially sensitive data. Image preprocessing includes resizing and normalization to match model input requirements.

Basic error handling is implemented to manage missing models, invalid inputs, and inference-time failures. In the event of upload or processing errors, the API returns informative error messages to the client.

The current implementation is intended for research and prototyping purposes. Future extensions will incorporate authentication, encrypted communication, structured logging, and healthcare.

Each deep learning model operates as an independent module callable through standardized API endpoints (/predict/cnn, /predict/vgg19, /predict/xception). This modularity allows seamless integration of additional models or tasks (e.g., segmentation, detection) in the future without re-engineering the whole platform architecture. Table 13 further highlights the API endpoints used.

**Table 13:** API endpoints

Endpoint	Method	Description
/tasks	GET	Returns available tasks and clinical objectives
/models/{task}	GET	Returns available models for the selected task
/predict	POST	Accepts image upload and metadata (task, clinical objective, model) and returns prediction

### Scalability and deployment considerations

Although the system architecture is designed to be extensible, formal scalability and load testing were not conducted in this study. The current implementation should therefore be considered a single-user research prototype rather than a production-ready system.

Model inference is performed synchronously using a single FastAPI instance with in-memory model caching. While this design reduces inference latency for individual requests, throughput under concurrent access (e.g., multiple simultaneous uploads) was not evaluated.

Comprehensive scalability assessment, including stress testing under concurrent user loads and deployment with asynchronous request handling or container orchestration, is planned as future work.

### Interface validation

The web interface was internally validated by the authors, Dr. Hadi Fayad and Dr. Tarraf Torfeh, who are medical physicists.

## Conclusion and perspectives

This study presents the development of a modular AI-powered medical imaging platform for brain tumor MRI classification that integrates a custom CNN with two pretrained deep learning models (VGG19 and Xception) in a single web-accessible interface. By combining standardized preprocessing, comparative benchmarking, and a user-friendly front-end, the system enables non-programmers to upload T1-weighted contrast-enhanced MRI slices and obtain fast, automated multi-class predictions (glioma, meningioma, pituitary, and no tumor). The experimental results show that the VGG19-based transfer-learning model achieved the best overall performance, with a test accuracy of 98% and balanced precision, recall, and F1-score, while the custom CNN and Xception models also delivered competitive results. These findings are consistent with recent work demonstrating that deep learning–based oncologic imaging pipelines can achieve high diagnostic performance while emphasizing the need for clinically oriented workflows that bridge algorithm development and patient care. Aside from just the performance metrics, the real value of this work is found in its adaptability and architectural design. In other words, this project bridges the gap between deep learning research and clinical application by emphasizing system integration and usability, in contrast to many previous studies that only concentrate on model training and evaluation. Because of the platform’s modular design, researchers and clinicians can add new diagnostic tasks, alter preprocessing pipelines, and even add in more AI models without having to start from scratch. This adaptability encourages experimentation, as well as the tool’s long-term use with all the new algorithms and imaging modalities being developed every now and then [50].

### Limitations & risk analysis

This study has several important limitations that must be acknowledged. The most critical limitation is the lack of external validation, as all models were trained and evaluated on a single public, slice-based dataset containing approximately 7,000 MRI images across four classes, without patient-level identifiers. Slice-level evaluation may inflate performance by introducing information leakage across slices from the same patient and limits conclusions regarding real-world generalizability. This introduces a risk that performance may decrease when applied to independent patient-level or multi-center clinical datasets.

From a data perspective, the dataset does not reflect true clinical prevalence, as class distributions are more balanced than typically observed in practice. In addition, the use of curated Kaggle data may introduce selection bias, as images are generally of high quality and may not capture rare tumor types, atypical presentations, or acquisition artifacts commonly encountered in clinical settings. These factors introduce a risk of reduced robustness when confronted with more heterogeneous real-world data.

Methodologically, the use of 2D slices prevents modeling of volumetric tumor characteristics, and only T1-weighted contrast-enhanced images were considered, whereas clinical diagnosis relies on multi-sequence MRI. The absence of data augmentation and patient-level cross-validation may further contribute to optimistic performance estimates. Observed misclassifications between tumor classes with overlapping imaging features also indicate a potential risk of diagnostic error, particularly in borderline or ambiguous cases.

In addition, due to computational constraints, repeated validation was performed only for the baseline CNN, while the deeper transfer-learning models were evaluated using a single split. Although this design ensures feasibility, repeated evaluation of all architectures could provide a more comprehensive comparison of performance stability. This may introduce uncertainty regarding model robustness across alternative data partitions.

From a clinical translation standpoint, no comparison with radiologist performance was conducted, and no formal usability or cost-effectiveness analyses were performed. Additionally, computational requirements and infrastructure constraints may limit deployment in resource-limited environments. From a risk perspective, false negatives or class confusion in a clinical setting could contribute to delayed or inaccurate decision support, underscoring the need for validation prior to deployment.

Future work will address these limitations through patient-level and multi-center external validation, incorporation of multi-sequence and 3D inputs, advanced regularization and augmentation strategies, clinician-in-the-loop evaluation, and systematic assessment of clinical utility and deployment feasibility.

### Future work

Looking at it from a wider angle, this project supports the integration of AI into the broader healthcare ecosystem by emphasizing modularity, scalability, and future extensibility. For example, incorporating multi-task learning approaches that combine tumor segmentation and classification, potentially leveraging seminal datasets such as the BraTS challenge to improve tumor boundary delineation and feature representation [51]. It also highlights key challenges that remain before routine clinical deployment, including multi-center validation, robustness to heterogeneous acquisition protocols, and alignment with local regulatory and governance frameworks. Future iterations of this platform should therefore follow emerging guidelines for clinical evaluation and reporting of AI decision-support tools, such as the DECIDE-AI framework [52], STARD-AI [53], and SPIRIT-AI [54] for early-stage, live clinical studies, and move toward prospective, multi-center trials that assess real-world impact, safety, and human–AI interaction.

For AI systems used in diagnostic imaging, explainability is essential for clinician trust and regulatory approval. Although the current implementation focuses primarily on classification accuracy, the platform is designed to integrate explainability modules such as Grad-CAM or Score-CAM.

These tools can highlight tumor-relevant regions, enabling radiologists to verify whether the model focuses on clinically meaningful anatomical structures. This is particularly important for gliomas and meningiomas, where subtle differences guide treatment planning.

The integration of explainability aligns with emerging guidelines such as DECIDE-AI and FDA GMLP, which require interpretable outputs in early-stage clinical AI evaluation. Future work will systematically evaluate the clinical usefulness of these explanations in collaboration with radiologists.

Ultimately, this work provides, a step toward the smooth transition of deep learning models from the lab to real healthcare settings. By exposing multiple architectures within a single, flexible web environment, it demonstrates how AI-based brain tumor classifiers can be made more transparent, testable, and clinically relevant. In the longer term, extending this framework to additional tumor types, imaging sequences, and multimodal data, while incorporating rigorous clinical evaluation, could help realize the full potential of AI-assisted neuro-oncology and support timelier, data-driven decisions for patients with brain tumors.

## Data Availability

All data generated or analyzed during this study are included in this published article. The link of the public MRI dataset that is used in this study is: [https://www.kaggle.com/datasets/masoudnickparvar/brain-tumor-mri-dataset].

## Code availability

Code will be available upon request. Trained model weights: https://github.com/jennifer-hamalaya/medical-ai-platform.git. Web demo: https://youtu.be/67VGnK6-CXg.

## References

[1] Beach CZ, Febres-Aldana CA, Marti JLG, et al. Clinical and preclinical insights into a novel MDM2::PDGFRA fusion in recurrent glioblastoma. NPJ Precis Oncol. 2025;9:289. 10.1038/s41698-025-01076-4. PMC12357959 40819143

[2] Sipos D, Raposa BL, Freihat O, Simon M, Mekis N, Cornacchione P, Kovács Á. Glioblastoma: Clinical presentation, multidisciplinary management, and long-term outcomes. Cancers (Basel). 2025;17(1):146. 10.3390/cancers17010146. PMC11719842 39796773

[3] Alruwaili AA, De Jesus O. Meningioma. In: StatPearls [Internet]. Treasure Island (FL): StatPearls Publishing; 2025 Jan [updated 2023 Aug 23]. Available from: https://www.ncbi.nlm.nih.gov/books/NBK554556/.

[4] Russ S, Anastasopoulou C, Shafiq I. Pituitary adenoma. In: StatPearls [Internet]. Treasure Island (FL): StatPearls Publishing; 2025 Jan [updated 2023 Mar 27]. Available from: https://www.ncbi.nlm.nih.gov/books/NBK538164/.

[5] Podstawka M, Dębska A, Szmyd B, Zaczkowski K, Piotrowski M, Bobeff EJ, et al. Advancing precision in neuro-oncology with intraoperative imaging and fluorescence guidance: a narrative review. Biomedicines. 2025;13:2550. 10.3390/biomedicines13102550 PMC12561074 41153830

[6] Parillo M, Mallio CA, Pileri M, Dirawe D, Romano A, Bozzao A, Weinberg B, Quattrocchi CC. Interrater reliability of Brain Tumor Reporting and Data System (BT-RADS) in the follow up of adult primary brain tumors: a single institution experience in Italy. Quant Imaging Med Surg. 2023;13(11):7423–31. 10.21037/qims-22-850. PMC10644140 37969622

[7] Al Yassin A, Salehi Sadaghiani M, Mohan S, Bryan RN, Nasrallah I. It is about time: Academic neuroradiologist time distribution for interpreting brain MRIs. Acad Radiol. 2018;25(12):1521–5. 10.1016/j.acra.2018.04.014. 29929936

[8] Pinto-Coelho L. How artificial intelligence is shaping medical imaging technology: a survey of innovations and applications. Bioengineering. 2023;10(12):1435. 10.3390/bioengineering10121435 PMC10740686 38136026

[9] Saeedi S, Rezayi S, Keshavarz H, Niakan Kalhori SR. MRI-based brain tumor detection using convolutional deep learning methods and selected machine learning techniques. BMC Med Inf Decis Mak. 2023;23:16. 10.1186/s12911-023-02114-6 PMC9872362 36691030

[10] Abdusalomov AB, Mukhiddinov M, Kutlimuratov A, Whangbo T. Brain tumor detection based on deep learning approaches and magnetic resonance imaging. Cancers (Basel). 2023;15(16):4172. 10.3390/cancers15164172 PMC10453020 37627200

[11] Yadav AC, Shah K, Purohit A, Kolekar MH. Computer-aided diagnosis for multi-class classification of brain tumors using CNN features via transfer-learning. Multimed Tools Appl. 2025;84(31):38959–38982. doi:10.1007/s11042-025-20751-z. Available from: 10.1007/s11042-025-20751-z.

[12] Yadav AC, Kolekar MH. Deep feature-based approaches for brain tumor classification and segmentation in medical imaging. Biomed Signal Process Control. 2026;117:109603.

[13] Cardoso MJ, Li W, Brown R, Ma N, Kerfoot E, Wang Y, et al. MONAI: An open-source framework for deep learning in healthcare. arXiv [Preprint]. 2022. Available from: https://arxiv.org/abs/2211.02701.

[14] Nasarian E, Alizadehsani R, Acharya UR, Tsui KL. Designing interpretable ML system to enhance trust in healthcare: a systematic review to proposed responsible clinician-AI-collaboration framework. arXiv [Preprint]. 2023. Available from: https://arxiv.org/abs/2311.11055.

[15] Raheem M, Elgammal A, Papazoglou M, Krämer B, El-Tazi N. A model-driven engineering approach to AI-powered healthcare platforms. arXiv [Preprint]. 2025. Available from: https://arxiv.org/abs/2510.09308.

[16] Food and Drug Administration (FDA), Health Canada, Medicines and Healthcare products Regulatory Agency (MHRA). Transparency for machine learning-enabled medical devices: guiding principles. 2024. Available from: https://www.fda.gov/medical-devices/software-medical-device-samd/transparency-machine-learning-enabled-medical-devices-guiding-principles.

[17] Satushe V, Vyas V, Metkar S, Singh DP. AI in MRI brain tumor diagnosis: a systematic review of machine learning and deep learning advances (2010–2025). Chemom Intell Lab Syst. 2025;263:104563.

[18] Sevak MM, Dwivedi VJ, Patel SS, Pandya R, Shah VV. Brain tumor detection using ResNet architectures. In: 2023 3rd International Conference on Intelligent Technologies (CONIT). 2023. p. 1–5.

[19] Wang D, et al. Multi-label classification of retinal disease via a novel vision transformer model. Front Neurosci. 2024;17:1101234. 10.3389/fnins.2023.1290803 PMC10800810 38260025

[20] Lalinia M, Sahafi A. Colorectal polyp detection in colonoscopy images using YOLO-v8 network. Signal Image Video Process. 2023;17:345–56.

[21] Christabel S, et al. An efficient DenseNet for diabetic retinopathy screening. Int J Eng Technol Innov. 2023;13(2):125–36.

[22] Kumaran SD, et al. Explainable lung cancer classification with ensemble transfer learning of VGG16, ResNet50, and InceptionV3 using Grad-CAM. BMC Med Imaging. 2024;24:45. 10.1186/s12880-024-01345-x PMC11264852 39030496

[23] Khalighi S, Reddy K, Midya A, Pandav KB, Madabhushi A, Abedalthagafi M. Artificial intelligence in neuro-oncology: advances and challenges in brain tumor diagnosis, prognosis, and precision treatment. NPJ Prec Oncol. 2024;8:80. 10.1038/s41698-024-00575-0. PMC10980741 38553633

[24] Obuchowicz R, Lasek J, Wodziński M, Piórkowski A, Strzelecki M, Nurzynska K. Artificial intelligence-empowered radiology—current status and critical review. Diagnostics (Basel). 2025;15(3):282. 10.3390/diagnostics15030282. PMC11816879 39941212

[25] Vysioneer Inc. VBrain: first-ever AI-powered tumor auto-contouring solution receives U.S. Food and Drug Administration clearance for radiation therapy. PR Newswire. 2021 Apr 5. Available from: https://www.prnewswire.com/news-releases/vysioneer-receives-fda-clearance-on-first-ever-tumor-auto-contouring-solution-for-radiation-therapy-301262201.html.

[26] MRIMath LLC. MRIMath LLC, a medical AI company, announces FDA 510(k) clearance for its cyber device and AI solution for glioblastoma. Business Wire. 2024 Aug 21. Available from: https://www.businesswire.com/news/home/20240820135568/en/MRIMath-LLC-a-Medical-AI-Company-Announces-FDA-510k-Clearance-for-its-Cyber-Device-and-AI-Solution-for-Glioblastoma.

[27] Cortechs.ai. Cortechs.ai announce next-gen NeuroQuant^®^ Brain Tumor with AI-driven metastasis and meningioma segmentation following FDA clearance. PR Newswire. 2025 Jul 30. Available from: https://www.prnewswire.com/news-releases/cortechsai-announce-next-gen-neuroquant-brain-tumor-ai-driven-metastasis-meningioma-segmentation-302516471.html.

[28] Aksoy S, Demircioglu P, Bogrekci I. A web-deployed, explainable AI system for comprehensive brain tumor diagnosis. Neurol Int. 2025;17(8):121. 10.3390/neurolint17080121 PMC12388561 40863990

[29] Haque R, Khan MA, Rahman H, Khan S, Siddiqui MIH, Limon ZH, Swapno SMMR, Appaji A. Explainable deep stacking ensemble model for accurate and transparent brain tumor diagnosis. Comput Biol Med. 2025;191:110166. 10.1016/j.compbiomed.2025.110166. 40249992

[30] Rastogi D, Johri P, Donelli M, Kumar L, Bindewari S, Raghav A, Khatri SK. Brain tumor detection and prediction in MRI images utilizing a fine-tuned transfer learning model integrated within deep learning frameworks. Life (Basel). 2025;15(3):327. 10.3390/life15030327. PMC11944010 40141673

[31] Nickparvar M. Brain tumor MRI dataset [Internet]. Kaggle. 2021 Sep 24. Available from: https://www.kaggle.com/datasets/mateuszbuda/lgg-mri-segmentation.

[32] Rasheed Z, Ma YK, Ullah I, et al. Brain tumor classification from MRI using image enhancement and convolutional neural network techniques. Brain Sci. 2023;13(9):1320. 10.3390/brainsci13091320 PMC10526310 37759920

[33] Mohsen S, Oraby S, Abdel-Aziz M. Deep learning and machine learning for brain tumor detection: a review, challenges, and future directions. Arch Comput Methods Eng. 2025;32:145–67.

[34] Mohammed FA, Tune KK, Assefa BG, Jett M, Muhie S. Medical image classifications using convolutional neural networks: a survey of current methods and statistical modeling of the literature. Mach Learn Knowl Extr. 2024.

[35] Krizhevsky A, Sutskever I, Hinton GE. ImageNet classification with deep convolutional neural networks. Adv Neural Inform Process Syst (NeurIPS). 2012;25:1097–105.

[36] Goodfellow I, Bengio Y, Courville A. Deep learning. Cambridge (MA): MIT Press; 2016. Chapter 6. Available from: https://www.deeplearningbook.org.

[37] Simonyan K, Zisserman A. Very deep convolutional networks for large-scale image recognition. In: International Conference on Learning Representations (ICLR). 2015. Available from: https://arxiv.org/abs/1409.1556.

[38] Lin M, Chen Q, Yan S. Network in network. arXiv [Preprint]. 2013. arXiv:1312.4400.

[39] Chollet F. Xception: deep learning with depthwise separable convolutions. In: proceedings of the IEEE conference on computer vision and pattern recognition (CVPR). 2017.

[40] US Food and Drug Administration. Good machine learning practice for medical device development: guiding principles [Internet]. FDA; 2021 Oct [updated 2025 Dec 19]. Available from: https://www.fda.gov/medical-devices/software-medical-device-samd/good-machine-learning-practice-medical-device-development-guiding-principles.

[41] Dehbozorgi P, Ryabchykov O, Bocklitz T. A systematic investigation of image pre-processing on image classification. IEEE Access. 2024;12:1–13.

[42] Shahamiri AR, et al. Negligible effect of brain MRI data preprocessing for tumor segmentation. Biomed Signal Process Control. 2024;94:106281.

[43] Islam MS, et al. Comparison of fine-tuning strategies for transfer learning in medical image classification. Image Vis Comput. 2024;145:105003.

[44] Khan MGRN, Islam MM, Uddin MS. Comparison of simple augmentation transformations for a convolutional neural network classifying medical images. SIViP. 2024;18:1–11.

[45] Sathya R, et al. Employing Xception convolutional neural network through high-precision MRI analysis for brain tumor diagnosis. Front Med. 2024;11:1487713. 10.3389/fmed.2024.1487713 PMC11601128 39606635

[46] Patel S, Sharma R. Impact of data augmentation on the performance of CNN models. Int J Adv Comput Sci Appl. 2025.

[47] Aly AA, Karar ME, Aboalsamh HA. Deep learning in medical image analysis: recent advances, challenges, and future directions. Semin Ultrasound CT MR. 2025;46(2):101–118.

[48] Shorten H, Khoshgoftaar TM. A survey on image data augmentation for deep learning. J Imaging. 2023;9(4):81.

[49] Disci R, Gurcan F, Soylu A. Advanced brain tumor classification in MR images using transfer learning and pre-trained deep CNN models. Cancers (Basel). 2025;17:1123. 10.3390/cancers17010121 PMC11719945 39796749

[50] Huang J, Xiang Y, Gan S, et al. Application of artificial intelligence in medical imaging for tumor diagnosis and treatment: a comprehensive approach. Discov Oncol. 2025;16:1625. 10.1007/s12672-025-03307-3 PMC12381339 40856916

[51] Menze BH, Jakab A, Bauer S, et al. The Multimodal Brain Tumor Image Segmentation Benchmark (BRATS). IEEE Trans Med Imaging. 2015;34(10):1993–2024. 10.1109/TMI.2014.2377694. PMC4833122 25494501

[52] Vasey B, Nagendran M, Campbell B, et al. DECIDE-AI expert group. Reporting guideline for the early stage clinical evaluation of decision support systems driven by artificial intelligence: DECIDE-AI. BMJ. 2022;377:e070904. 10.1136/bmj-2022-070904 PMC9116198 35584845

[53] Sounderajah V, Guni A, Liu X, et al. The STARD-AI reporting guideline for diagnostic accuracy studies using artificial intelligence. Nat Med. 2025;31:3283–9. 10.1038/s41591-025-03953-8. 40954311

[54] Rivera SC, Liu X, Chan A-W, SPIRIT-AI and CONSORT-AI Working Group, et al. Guidelines for clinical trial protocols for interventions involving artificial intelligence: the SPIRIT-AI Extension. BMJ. 2020;370:m3210. 10.1136/bmj.m3210. PMC7490785 32907797

